# Understanding anaerobic germination in direct-seeded rice: a genomic mapping approach

**DOI:** 10.1186/s12870-024-05901-z

**Published:** 2024-12-19

**Authors:** Vikas Kumar Verma, Nitika Sandhu

**Affiliations:** Punjab Agricultural University, Punjab, Ludhiana 141004 India

**Keywords:** Anaerobic germination, Direct-seeded rice, Genome wide association studies, Marker-trait associations

## Abstract

**Background:**

Anaerobic germination is a critical trait for rice cultivation, particularly in regions that experience flooding or waterlogging immediately after sowing. Under direct-seeded conditions, where rice is sown directly into the field without prior transplantation, the ability of seeds to germinate in anaerobic (oxygen-deficient) conditions becomes essential for successful crop establishment. This trait is especially relevant in areas prone to waterlogging, were traditional methods of rice cultivation, such as puddled transplanting, may be less viable. Understanding the genetic basis of anaerobic germination can lead to the development of rice varieties that are better adapted to such challenging conditions, thus supporting more sustainable agricultural practices.

**Results:**

In this study, a nested association mapping (NAM) population consisting of 384 breeding lines was utilized to identify genomic regions associated with anaerobic germination in rice. Through comprehensive analysis, 19 significant marker-trait associations (MTAs) were identified, including 12 associations specifically linked to percent seed germination under anaerobic conditions. These associations were distributed across six different chromosomes: 3, 4, 5, 6, 7, and 9. Notably, a cluster of single nucleotide polymorphisms (SNPs) spanning a 6.9 Mb genomic region on chromosome 3 (from 21,089,181 to 28,017,712 bp) was consistently associated with percent germination at 15 and 21 days after sowing over multiple years. Similarly, a 6.4 Mb genomic segment on chromosome 6 (from 18,028,538 to 24,492,161 bp) was also associated with percent germination at the same time points. Specific SNPs within this region, namely S6_18028538 and S6_24492161, were linked to germination at 15 and 21 days, respectively. In addition to these findings, one MTA was identified for days to 50% flowering on chromosome 1, and six MTAs were identified for grain yield across chromosomes 1, 2, 5, 8, and 10. The breeding lines that exhibited both high and stable yields, along with anaerobic germination traits, have the potential to be particularly valuable in genomics-assisted breeding programs aimed at improving rice varieties for flood-prone areas.

**Conclusions:**

This study provides crucial insights into the genetic basis of anaerobic germination in rice, highlighting specific genomic regions associated with this trait under direct-seeded conditions. The identification of significant MTAs across multiple chromosomes, particularly the consistent associations found on chromosomes 3 and 6, underscores the potential for developing rice varieties with enhanced tolerance to anaerobic conditions. The high-yielding breeding lines identified in this research, which also exhibit strong anaerobic germination traits, represent valuable genetic resources for breeding programs. These findings support the use of direct-seeded rice (DSR) as a sustainable alternative to traditional puddled transplanting, particularly in regions prone to flooding, thereby contributing to the development of more resilient rice cultivation practices.

**Supplementary Information:**

The online version contains supplementary material available at 10.1186/s12870-024-05901-z.

## Introduction

Rice (*Oryza sativa* L.) occupies an important place in Indian agriculture and forms the staple diet of no less than 70% of the population [1]. It is an integral part of India, both in terms of production and trade, but being a water guzzling crop its negative impact on natural resources is a major concern. In view of the urgency to conserve groundwater resources on one hand and maintain food security as well as farmer’s livelihood on the other, technologies aimed at saving water in rice cultivation hold significance. Direct Seeded Rice (DSR) is one such promising technology with water and labor-saving possibilities. Additionally, it solves several other problems as it involves less drudgery, matures early, lowers production costs, provides better soil conditions, and reduces methane emissions [2–5]. Short duration rice varieties have shown encouraging results under DSR conditions and are gaining popularity amongst farmers. However, these varieties being used for DSR have been basically developed for puddled transplanted rice (PTR) and lack several of the attributes required for better adaptation to direct seeding. Serious problems inherent to use of conventional rice varieties for direct seeding, such as poor germination under anaerobic conditions and inability of seed to emerge from depth, higher incidence of brown spot, nematode infestation, iron deficiency under light soils and poor milling quality pose a challenge to wider adoption and success of DSR.

Anaerobic germination is one among the most important traits for direct seeded rice (DSR) cultivation conditions. Anaerobic germination enables seedlings to emerge even when submerged in water, contributing significantly to better crop stand establishment. In regions affected by erratic monsoons, poor field leveling, and inadequate drainage, the unpredictability of water levels can lead to uneven crop stands and poor establishment of rice plants. However, rice genotypes equipped with the trait of anaerobic germination have the capacity to germinate and emerge even above the water surface, offering a significant advantage in such conditions. The wide adoption of DSR is majorly hindered by the incidence of rain within 4–5 days of sowing, causing anaerobic environment for germinating seeds/seedlings or resulting increase in depth of sowing with soil flowing into the seeded furrow with rain water. An ideal plant type for DSR should have the ability to germinate under anaerobic condition coupled with tolerance of early submergence and good seedling vigor. This may play a pivotal role in promoting the widespread adoption of direct seeded rice cultivation [6]. This trait not only ensures better crop stand establishment but also enhances the resilience of rice cultivation in areas prone to flooding or where water levels fluctuate, ultimately contributing to increased productivity and sustainability in rice farming practices.

The physiological and molecular mechanism underlying germination of rice under anaerobic condition is very complex and involves various processes such as the breakdown of starch, glycolysis, fermentation, increased activity of enzymes, and several other biochemical and metabolic processes besides hormone signaling [6, 7]. In recent years, extensive efforts have been made in harnessing the genetic basis of anaerobic germination in rice exploring quantitative trait loci (QTL) mapping and genome-wide association studies (GWAS) [8–16].

GWAS is a popular and highly efficient mapping strategy for dissecting the complex traits, but very few reports regarding the mine of genetic loci associated with anaerobic germination under DSR via GWAS are available. Using 5291 SNPs and 432 *indica* varieties in GWAS, a total of 15 anaerobic germination tolerance loci were detected [17]. As the best of our knowledge, till date only one QTL (qAG-9-2) has been fine mapped and cloned as *OsTPP7* [14]. Under anaerobic conditions, the *OsTPP7* gene increased the turnover of trehalose-6-phosphate, thus enhancing the mobilization of starch and driving the growth kinetics of germinating embryo resulted in anaerobic germination tolerance. Though, the exact mechanism of tolerance to anaerobic germination is still not fully understood and warrants further investigation [18]. Molecular breeding efforts on rice for improving tolerance to the anaerobic conditions during germination and seedling vigor have been attempted in past [19–21]. The availability of limited knowledge underlying the genetics of the trait, involvement of complex mechanism and lack of an effective screening method are among the reasons of slow progress achieved over the past few years [9]. Therefore, a large gap between the identification of AG tolerance genes and the breeding of DSR rice varieties still exists.

Researchers at International Rice Research Institute (IRRI) identified donors for anaerobic germination including IR14D155, IR15D120, IR13F474, IR10F365, IR 127152-2-10, IR 127155-1-22, IR 93312-30-101-20-13-66-6 [10, 11, 22, 23]. Hence, breeding population derived from the donor possessing the ability to germinate under anaerobic conditions will help in dissection of genomic region associated with anaerobic germination trait. The present study aims to contribute to this work by evaluating a set of breeding lines from the ongoing DSR breeding programme, generated from crosses involving seven donor parents possessing anaerobic germination trait with well adapted popular rice cultivar, PR126 possessing productivity and quality traits. Attempts will be made to harness the genomic regions associated with improved germination under anaerobic conditions in DSR. This will help us to identify the putative genes involved and investigate their differential expression further facilitating the breeding of rice cultivars suitable for direct seeded conditions.

## Results

### Development of mapping population

**Fig. 1:**
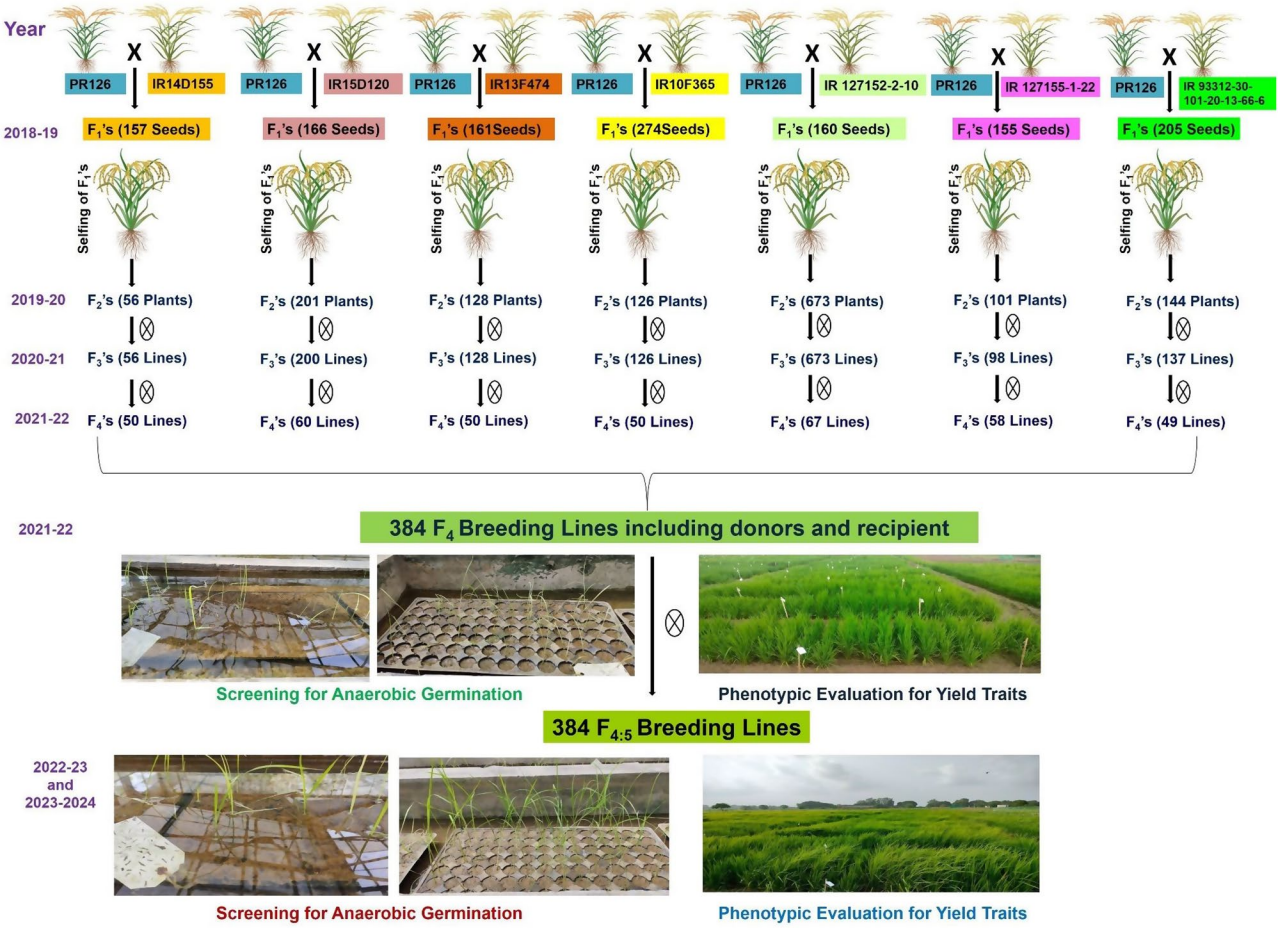
The picture representing comprehensive details on the crossing strategy and the number of plants selected across various generations and years to create the nested association mapping population

The seven accessions (IR14D155, IR15D120, IR13F474, IR10F365, IR 127152- 2-10, IR 127155-1-22, IR 93312-30-101-20-13-66-6) for anaerobic germination were used as donor parents and the PR126, most cultivated variety of Punjab was used as recipient parent. The detailed information on the crossing strategy and number of plants across generations is presented in Fig. 1. A total of 384 breeding lines derived from seven sub-populations (PR126/IR14D155: 50 breeding lines; PR126/IR15D120: 60 breeding lines; PR126/IR13F474: 50 breeding lines; PR126/IR10F365: 50 breeding lines; PR126/IR 127152-2-10: 67 breeding lines; PR126/IR 127155-1-22: 58 breeding lines; PR126/IR 93312-30-101-20-13-66-6: 49 breeding lines) were used in the present study. These breeding lines at F_4_, F_5_ and F_6_ generations were screened for anaerobic germination and other morphological/agronomic traits during *Kharif* season 2021, 2022 and 2023 under controlled conditions in glasshouse and field conditions, respectively.

### Standardization of phenotyping for anaerobic germination

**Fig. 2:**
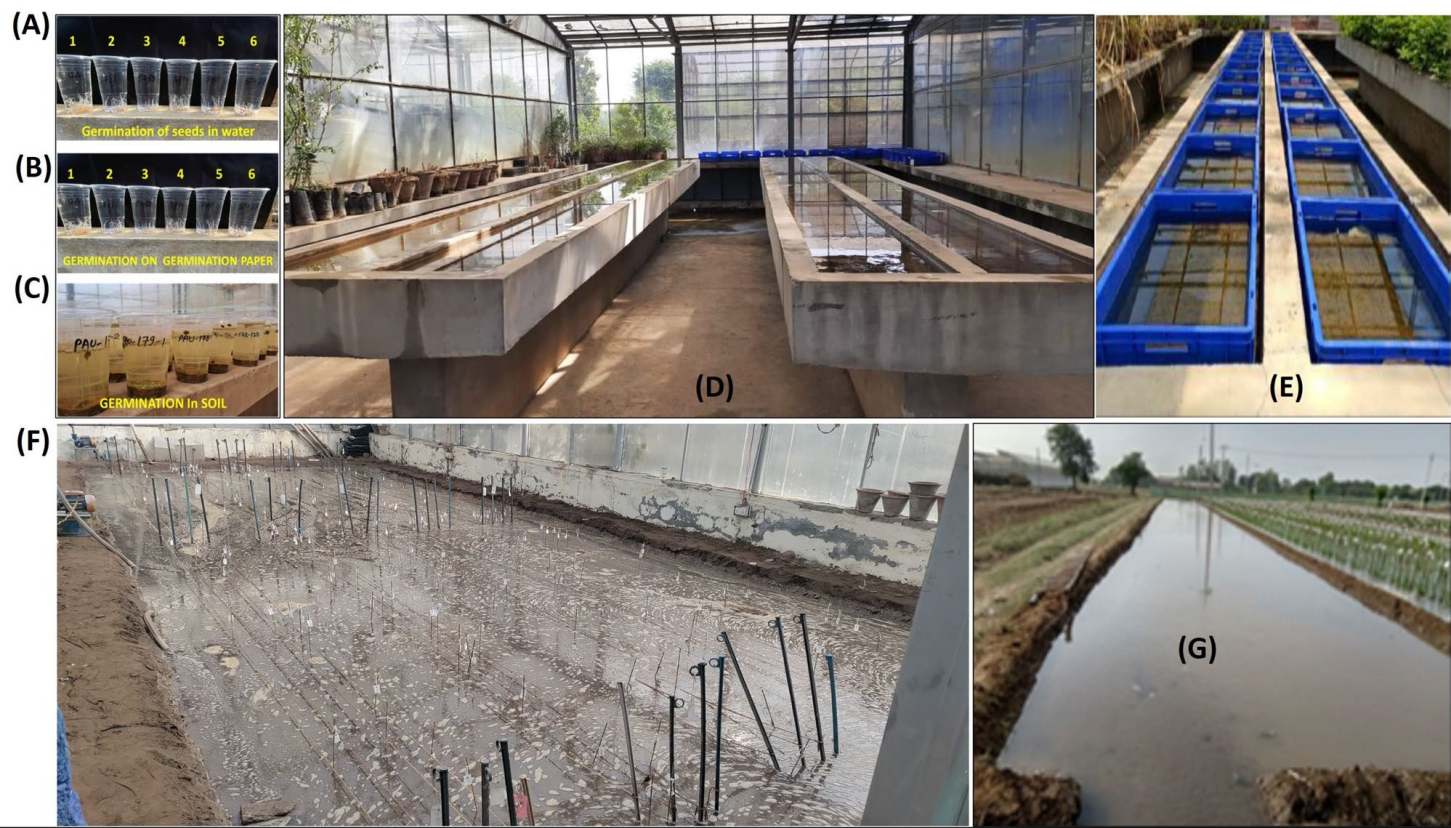
The pictorial representation of distinct experiments that were conducted to simulate anaerobic germination (**A**) anaerobic germination with water in plastic cups, (**B**) anaerobic germination with water and germination paper in plastic cups (**C**) anaerobic germination with soil and water in plastic cups (**D**) anaerobic germination in plug trays filled with field soil placed on a concrete screening table, (**E**) anaerobic germination in plug trays placed in plastic trays (each measuring 60 cm in length, 40 cm in width, and 15 cm in height), (**F**) anaerobic germination directly on the puddled soil of a screenhouse to provide more natural field conditions, and (**G**) anaerobic germination under natural field conditions

Seven different experiments were conducted to determine the most effective screening method for anaerobic germination (Fig. 2). These included (i) in water, using plastic cups (ii) in soil and water, using plastic cups (iii) in water and on germination paper, using plastic cups (iv) in plug trays with field soil, placed on a concrete screening table (v) in plug trays placed in plastic trays (60 cm x 40 cm x 15 cm) (vi) directly on puddled soil in a screenhouse (vii) under natural field conditions. The percentage germination of the tolerant checks was averaged and compared with that of the susceptible checks. Significant differences between the susceptible check and the average of the tolerant checks were observed across all experiments, except for experiments 1 and 3 (Fig. S1A). The phenotyping for anaerobic germination using soil and water in plastic cups, plug trays filled with field soil on a concrete screening table, and plastic trays was found to be comparable to screening under natural field conditions (Fig. S1B).

### Characterization of phenotypic traits

**Fig. 3:**
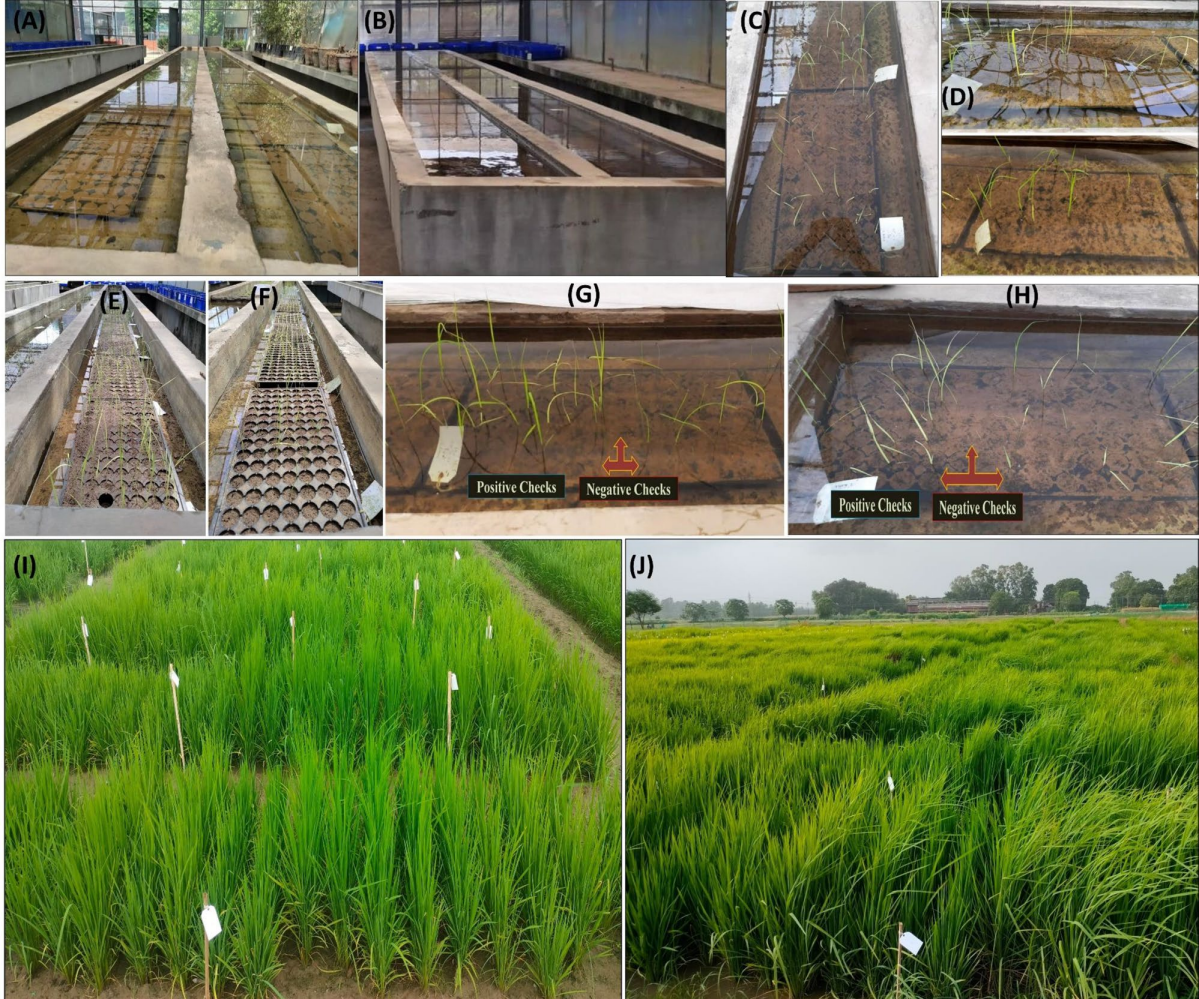
(**A**) The schematic representation of the experiments where water was maintained 15 cm above the plug tray surface for 15 days, (**B**) where water was maintained 15 cm above the plug tray surface for 21 days, (**C**) view of seedling germination after 15 days under anaerobic conditions, (**D**) view of seedling germination after 21 days under anaerobic conditions, (**E**) view of seedling survival when the water was removed after 15 days and the seeds were allowed to germinate for the next 10 days, (**F**) view of seedling survival when the water was removed after 21 days and the seeds were allowed to germinate for the next 10 days, (**G**) phenotypic variation in seedling germination of the positive (tolerant) and negative (susceptible) checks in the experiment where water was maintained 15 cm above the plug tray surface for 15 days, (**H**) phenotypic variation in seedling germination of the positive (tolerant) and negative (susceptible) checks in the experiment where water was maintained 15 cm above the plug tray surface for 21 days (**I**) Field view of the breeding lines for agronomic performance during the 2022 kharif season (**J**) Field view of the breeding lines for agronomic performance during the 2023 kharif season

Three sets of experiments including the experiments where (i) water was removed after 15 days, (ii) after 21 days and (iii) control were conducted to estimate the phenotypic variation in the nested association mapping population. The schematic representation of the experiments where the water was removed after 15 and 21 days is presented in Fig. 3A and H. A considerable degree of variations was observed across breeding lines in terms of their ability to survive in anaerobic germination conditions and the number of days required for germination under anaerobic conditions, as compared to positive checks. The positive checks exhibited germination starting from the seventh day, while the negative checks showed no anaerobic germination under water submerged condition (Fig. 3G and H). It was observed that the out of 384 breeding lines, a total of 296, 345 lines were germinated under anaerobic germination at 15th DAS during 2022 and 2023, respectively. These lines were classified into five categories based on germination percent under water submersed conditions. The anaerobic germination percent category includes 0, 1 to 25%, 26 to 50%, 51 to 75%, and 76 to 100%. The detailed information in the number of breeding lines in each category across years and experiments is presented in Table 1. The mean percentage germination at 15 DAS was 41.67%, 44.90% and 47.90% with higher heritability (h^2^) 82%, 81%, and 76% in 2021, 2022 and 2023 respectively (Table 2). Likewise, the mean percentage germination at 21 DAS was 31.60%, 25.74% and 38.80%, in 2021, 2022 and 2023, respectively (Table 2). Along with the anaerobic germination traits, the breeding lines were tested for agronomic performance under field conditions (Fig. 3I and J). The breeding lines showed better agronomic performance with overall mean performance for DTF 72 days, PHT 85 days, panicle length 23 cm, number of panicles/plant 9 and grain yield of 2072 kg ha^− 1^ across years (Table 2). The percent coefficient of variations (CV%) for the traits measured in the present study ranged from 2 to 18% across years.

**Table 1:** Population wise percent anaerobic germination across different years and treatments

Mapping populations	Percent Germination	%Ger15_2021	%Ger15_2022	%Ger15_2023	%Ger21_2021	%Ger21_2022	%Ger21_2023
PR126/IR14D155	0	17	15	14	12	18	14
	1–25	9	7	8	5	8	11
	26–50	8	9	8	9	14	14
	51–75	8	10	11	15	5	6
	76–100	8	9	9	9	5	5
PR126/IR15D120	0	12	14	10	8	12	17
	1–25	14	15	15	17	16	12
	26–50	12	13	16	13	12	13
	51–75	10	10	13	14	8	9
	76–100	12	8	6	8	12	9
PR126/IR13F474	0	15	12	18	18	15	10
	1–25	10	10	8	7	8	11
	26–50	10	10	6	11	9	7
	51–75	8	9	10	9	9	11
	76–100	7	9	8	5	9	11
PR126/IR10F365	0	15	12	10	6	11	9
	1–25	10	8	13	9	9	12
	26–50	15	15	10	14	10	8
	51–75	8	10	8	10	10	12
	76–100	2	5	9	11	10	9
PR126/IR127152-2-10	0	15	22	20	12	12	20
	0–25	21	10	10	19	14	12
	26–50	10	13	5	15	14	11
	51–75	14	10	19	11	11	15
	76–100	7	12	13	10	16	9
PR126/IR127155-1-22	0	12	15	101	15	12	10
	1–25	15	10	12	11	13	14
	26–50	13	15	13	15	12	11
	51–75	10	9	12	8	11	13
	76–100	8	9	10	9	10	10
PR126/IR13312-30- 101-20-13-66-6	0	10	19	12	5	15	18
	1–25	15	9	7	15	12	10
	26–50	5	10	10	12	8	6
	51–75	17	5	12	6	5	10
	76–100	2	6	8	11	9	5

%Ger15: Percentage of germination in the experiment where water was withdrawn 15 days after sowing; %Ger21: Percentage of germination in the experiment where water was withdrawn 21 days after sowing; 0, 1–25, 26–50, 51–75, 76–100: categories of percent germination under anaerobic conditions

**Table 2:** Detailed information on analysis of variance (ANOVA), mean, range and heritability of trait measured across years in different growing environments in NAM population and the mean comparison of the parental lines used to develop the NAM population

Env	Trait	Year	mean + SE	Std dev	CV (%)	PR126	IR14D155	IR15D120	IR13F474	IR10F365	IR 127152-2-10	IR 127155-1-22	IR 93312-30-101-20-13-66-6	Range	F value	***P*** Value	heritability (%)
Field	%Germination_15DAS	2021	41.67 ± 2.24	7.63	18.3	0.0 ± 3.17	73.1 ± 3.17	73.7 ± 3.17	80.2 ± 3.17	77.8 ± 3.17	84.3 ± 3.17	73.4 ± 3.17	79.3 ± 3.17	0.0-100.0	7.9835	*	82
Screenhouse		2022	44.90 ± 2.14	6.96	15.5	17.4 ± 3.18	78.1 ± 3.18	76.3 ± 3.18	84.2 ± 3.18	78.8 ± 3.18	86.3 ± 3.18	63.2 ± 3.18	67.3 ± 3.18	0.0-100.0	7.1923	*	81
Screenhouse		2023	47.9 ± 2.89	5.52	11.52	5.1 ± 2.20	75.4 ± 2.22	79.6 ± 2.33	81.5 ± 2.23	81.3 ± 2.87	82.3 ± 2.44	73.4 ± 1.33	75.2 ± 2.55	0.0-100.0	5.231	*	76
Screenhouse	%Germination_21DAS	2021	31.60 ± 2.01	5.16	16.34	6.13 ± 2.85	67.3 ± 2.85	63.4 ± 2.85	73.8 ± 2.85	60.8 ± 2.85	75.5 ± 2.85	70.9 ± 2.85	56.1 ± 2.85	0.0-100.0	7.6768	***	75
Screenhouse		2022	25.74 ± 2.31	4.33	16.81	8.04 ± 3.26	52.4 ± 3.26	67.2 ± 3.26	76.1 ± 3.26	57.0 ± 3.26	78.6 ± 3.26	58.3 ± 3.26	60.2 ± 3.26	0.0-87.5	5.1997	***	68
Screenhouse		2023	38.8 ± 1.76	4.81	12.40	6.11 ± 2.21	63.4 ± 2.83	70.2 ± 2.77	72.1 ± 2.23	67.0 ± 2.69	75.3 ± 2.11	65.3 ± 2.75	64.2 ± 3.11	0.0-91.4	7.224	***	70
Field	Days to 50% flowering (days)	2021	72.20 ± 0.61	1.46	2.03	77 ± 1.01	75 ± 1.01	76 ± 1.01	76 ± 1.01	77 ± 1.01	76 ± 1.01	77 ± 1.01	76 ± 1.01	58–99	3.4163	NS	-
		2022	72.44 ± 0.71	1.46	2.02	76 ± 1.08	73 ± 1.08	74 ± 1.08	75 ± 1.08	76 ± 1.08	73 ± 1.08	75 ± 1.08	76 ± 1.08	57–102	2.5359	NS	-
		2023	78.34 ± 0.59	1.58	2.18	81 ± 1.88	78 ± 1.88	81 ± 1.88	82 ± 1.88	80 ± 1.88	79 ± 1.88	78 ± 1.88	81 ± 1.88	63–112	1.668	*	64
	Plant height (cm)	2021	86.10 ± 1.81	2.09	2.43	89 ± 2.69	91 ± 2.69	98 ± 2.69	98 ± 2.69	90 ± 2.69	83 ± 2.69	84 ± 2.69	85 ± 2.69	68–110	0.6392	NS	-
		2022	82.05 ± 1.50	1.80	2.20	88 ± 2.20	84 ± 2.20	88 ± 2.20	89 ± 2.20	98 ± 2.20	84 ± 2.20	85 ± 2.20	83 ± 2.20	60–111	0.7311	NS	-
		2023	92.69 ± 2.786	1.89	2.16	94 ± 2.07	89 ± 2.07	94 ± 2.07	103 ± 2.07	102 ± 2.07	88 ± 2.07	88 ± 2.07	90 ± 2.07	68–125	1	NS	-
	Panicle length (cm)	2021	23.38 ± 0.33	0.54	2.31	23 ± 0.45	22.6 ± 0.45	22.4 ± 0.45	23.2 ± 0.45	23.0 ± 0.45	22.7 ± 0.45	23.7 ± 0.45	23.1 ± 0.45	17–30	1.7856	***	73
		2022	21.41 ± 0.34	0.51	2.39	22 ± 0.52	21.3 ± 0.54	20.8 ± 0.52	21.7 ± 0.52	20.0 ± 0.56	21.1 ± 0.58	21.1 ± 0.58	21.3 ± 54	13–27	4.1622	*	64
		2023	24.61 ± 0.65	1.06	4.31	25 ± 0.47	25.0 ± 0.47	26 ± 0.47	26 ± 0.47	23 ± 0.47	23 ± 0.47	25 ± 0.47	26 ± 0.47	18–30	1.4635	*	60
	Number of panicles/plants	2021	7.98 ± 0.33	0.53	6.69	8.0 ± 0.54	8.0 ± 0.54	8.0 ± 0.54	9.0 ± 0.54	8.0 ± 0.54	7.0 ± 0.54	8.0 ± 0.54	8.0 ± 0.54	4–19	2.2919	*	59
		2022	7.92 ± 0.51	0.61	7.65	9.0 ± 0.75	10.0 ± 0.75	7.0 ± 0.75	8.0 ± 0.75	6.0 ± 0.75	7.0 ± 0.75	8.0 ± 0.75	8.0 ± 0.75	2–15	0.6886	NS	-
		2023	12.0 ± 1.0	0.65	5.25	11.0 ± 0.91	13.0 ± 0.91	13.0 ± 0.91	13.0 ± 0.91	13.0 ± 0.91	12.0 ± 0.91	13.0 ± 0.91	12.0 ± 0.91	`4–20	1.7178	*	58
	Grain yield (kg/ha)	2021	2601.59 ± 144.92	318.25	12.23	2803 ± 218.34	2269 ± 218.34	2025 ± 218.34	1941 ± 218.34	1842 ± 218.34	1893 ± 218.34	1438 ± 218.34	1477 ± 218.34	452–6702	2.801	***	62
		2022	1113.68 ± 78.91	121.68	10.93	1227 ± 111.60	1289 ± 111.60	850 ± 111.60	1144 ± 111.60	751 ± 111.60	1007 ± 111.60	1141 ± 111.60	1048 ± 111.60	208–3375	1.4562	**	54
		2023	2223.21 ± 134.86	258.54	10.34	2544 ± 340.95	2544 ± 340.95	2448 ± 340.95	2998 ± 340.95	2457 ± 340.95	2000 ± 340.95	2121 ± 340.95	1982 ± 340.95	250–6800	1.4202	**	60

%Ger15: Percentage of germination in the experiment where water was withdrawn 15 days after sowing; %Ger21: Percentage of germination in the experiment where water was withdrawn 21 days after sowing h^2^: heritability, %: percentage, P value: * Significant at < 0.05 level, ** Significant at 0.01 level, *** Significant at < 0.001 level, SE: standard error, Std dev: standard deviation, CV (%): coefficient of variations (percentage)

### Characterization of genotypic data

**Fig. 4:**
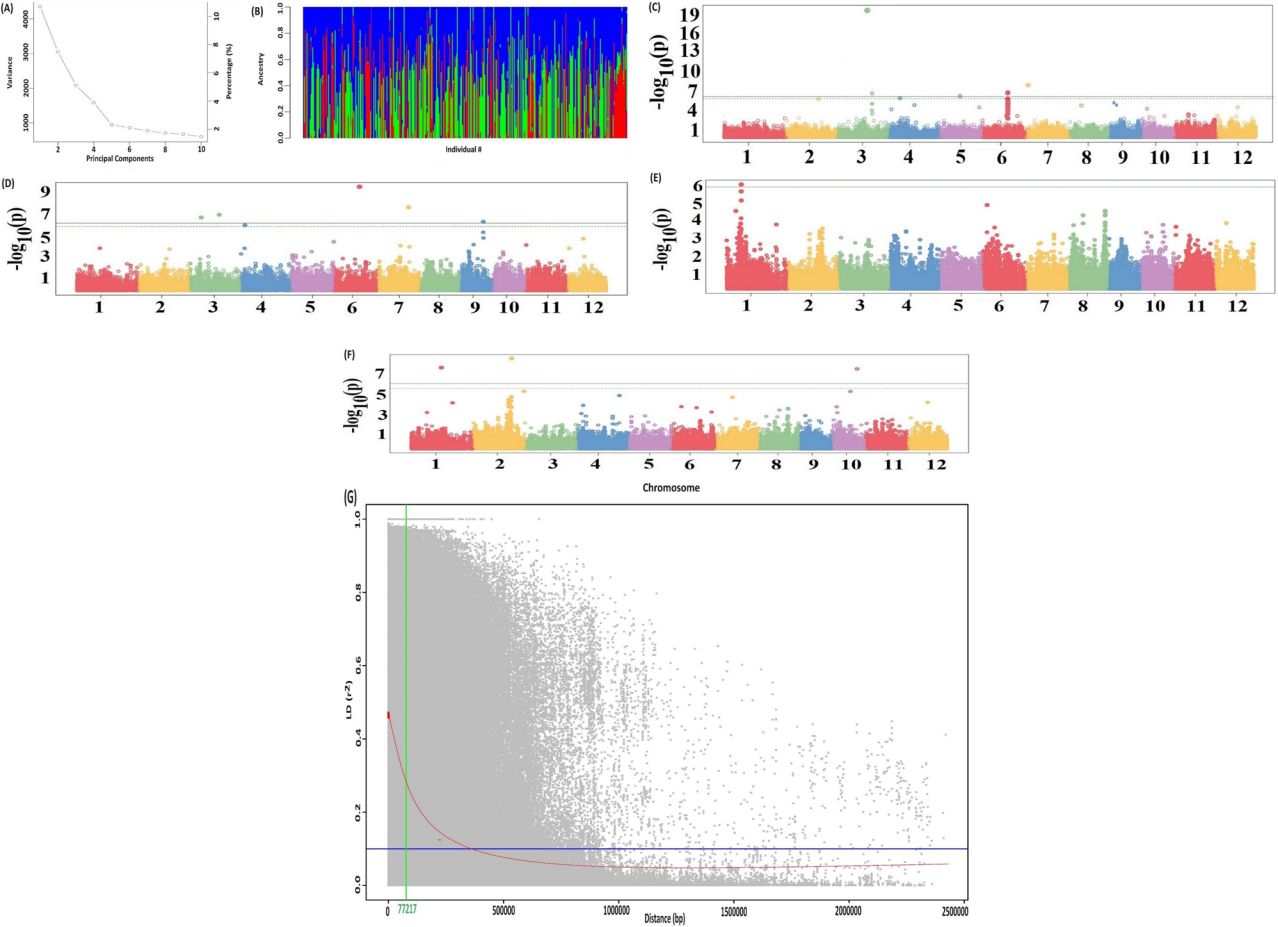
**(A)** The Scree plot indicating the most of the variability explained by first six Principal Components for association study **(B)** Population structure inferred by using the ADMIXTURE software. Each sample is denoted by a single vertical bar partitioned into K colors according to its proportion of ancestry in each of the clusters. Ancestral contributions for K = 6 are graphically represented **(C)** The manhattan plots depicting the significant -log (p-values) across years for the MTAs associated with percentage of germination in the experiment where water was withdrawn 15 days after sowing **(D)** The manhattan plots depicting the significant -log (p-values) across years for the MTAs associated with percentage of germination in the experiment where water was withdrawn 21 days after sowing **(E)** The manhattan plots depicting the significant -log (p-values) across years for the MTAs associated with days to 50% flowering **(F)** The manhattan plots depicting the significant -log (p-values) across years for the MTAs associated with grain yield **(G)** The scatterplot indicating the LD decay relationship between the r^2^ values on Y-axis and the genetic distance of the loci pairs (Mb) on X-axis

The ddRADseq analysis resulted in a total of 3,888,394 variants [SNPs + Indels]. Out of 3,888,394 variants, 3,604,027 SNPs and 3,578,039 biallelic SNPs were present. The dataset underwent data analysis and filtering procedures, which involved excluding SNPs with missing datapoints over 10%, minor allele frequencies (MAF) below 0.05, and read depths below 2 resulted in total of 79,933 SNPs. Finally, a total of 79,457 SNPs, the chromosome only SNP were used in the GWAS analysis. These SNPs were used to look at population structure using principal component analysis (PCA). The PCA and admixture analysis indicates that the first six principal components (PCs) were most informative and after that gradually decreasing until the tenth PC (Fig. 4A and B).

### Genome-wide association mapping

A total of 79,457 SNPs were used for GWAS to identify the causative loci underlying phenotypic variations. This study employed a GWAS methodology, utilizing a Farm CPU model that incorporated considerations for population structure and relative kinship. A total 19 marker traits associations (MTAs) were identified for anaerobic germination, days to 50% flowering and grain yield across years and the quantile-quantile (Q-Q) plot exhibited SNPs that displayed deviations from the anticipated distribution, potentially indicating an association with the observed feature. Similar to the FarmCPU approach, where the Bonferroni-corrected threshold is notably stringent, we also computed the threshold using the adjusted threshold function “p.threshold”. This method permutes the phenotypes to eliminate any spurious associations with the genotypes. Specifically, we employed 1000 permutations to assess the minimum p-value vector for each experiment conducted over multiple years. The 99% quantile value of this vector yielded the p.threshold, corresponding to a -log(p-value) of 4.35. Utilizing this -log(p-value) as a cutoff, we identified significant associations across experiments and years.

For % germination trait, a total of 11 MTAs covering 6 chromosomes (chromosome 3, 4, 5, 6, 7 and 9) including a cluster of significant SNPs on chromosome 3 were detected across years and experiments (Table 3; Fig. 4C and D). A 6.9 Mb genomic region extended from 21,089,181 bp to 28,017,712 bp constituting four significant SNPs on chromosome 3 reported to be associated with % germination at 15 and 21 days after sowing. The SNP S3_21089181 showed association with both the traits, % germination at 15 and 21 days after sowing. The 6.4 Mb genomic region extended from 1.80 Mb to 2.44 Mb encompassing 2 significant MTAs on chromosome 6 showed association with % germination at 15 and 21 days after sowing. The SNP S6_18028538 showed association with % germination at 21 days after sowing and the SNP S6_24492161 with % germination at 15 days after sowing. Further, the SNP S4_2823374 on chromosome 4, S5_14285254 on chromosome 5, S7_929082 and S7_21442267 on chromosome 7 and S9_15924439 on chromosome 9 associated with % germination trait were detected (Table 3). For days to 50% flowering, the SNP S1_10264431 on chromosome 1 displayed strong association (Fig. 4E). The GWAS for grain yield identified two significant SNPs at 23,945,779 bp and 26,444,161 bp on chromosome 2 (Fig. 4F). Similarly, the SNP S1_20943333 on chromosome 1, S5_1764039 on chromosome 5, SNP S8_10041964 on chromosome 8 and S10_17012689 on chromosome 10 showed significant association with grain yield (Fig. 4F).

**Table 3:** The significant marker-trait associations and putative candidate genes (https://plants.ensembl.org/biomart/martview) associated with anaerobic germination traits across years and experiments in a genome wide association study conducted on nested association mapping population under direct seeded cultivation conditions

Trait	SNP	Chr	Position (bp)	***P***.value	Effect	Function
%Germination_15DAS and 21DAS	S3_21089181	3	21,089,181	2.82E-06	8.667	fatty-acyl-CoA binding, RNA binding, polyamine transmembrane transporter activity, spindle microtubules to kinetochore, meiotic chromosome segregation, protein-containing complex binding, Ndc80 complex, cell cycle, cell division, UDP-glycosyltransferase activity
%Germination_15DAS	S3_24412222	3	24,412,222	2.47E-07	11.603	kinase activity, phosphorylation, sepal giant cell differentiation, multivesicular body membrane, cell surface, plant epidermal cell differentiation, flower morphogenesis, regulation of asymmetric cell division, ATP binding, endocytic vesicle, positive regulation of DNA endoreduplication, plant organ development, embryo development ending in seed dormancy, protein homodimerization activity, protein autophosphorylation, protein serine/threonine kinase activity, plasma membrane, transferase activity, membrane, nucleotide binding, endosome, vacuolar membrane, symporter activity, carbohydrate transport, transmembrane transporter activity
%Germination_21DAS	S3_25375913	3	25,375,913	1.31E-06	-4.747	nuclear-transcribed mRNA poly(A) tail shortening, CCR4-NOT core complex, P-body, regulation of DNA-templated transcription, nucleus, cytoplasm
%Germination_21DAS	S3_28017712	3	28,017,712	3.00E-06	-9.707	defence response, response to salt stress, hydrolase activity, sphingolipid metabolic process, acting on carbon-nitrogen (but not peptide) bonds, in linear amides, ceramide metabolic process, response to freezing and salt stress, seed oil body biogenesis, lipid storage, post-embryonic development, oxidoreductase activity
%Germination_21DAS	S4_2823374	4	2,823,374	9.82E-07	5.819	defence response, transferase activity, kinase activity
%Germination_15DAS	S5_14285254	5	14,285,254	5.93E-07	-9.487	transferase activity, cytokinin biosynthetic process, tRNA modification
%Germination_21DAS	S6_18028538	6	18,028,538	2.97E-10	9.914	chloroplast fission, transmembrane transporter activity, antiporter activity, aleurone grain nutrient reservoir activity
%Germination_15DAS	S6_24492161	6	24,492,161	5.60E-06	9.558	DNA binding, metal ion binding, DNA repair, cellular response to DNA damage stimulus, DNA-binding transcription factor activity, regulation of cellular biosynthetic process
%Germination_21DAS	S7_21442267	7	21,442,267	2.27E-08	-6.869	transferase activity, kinase activity, phosphorylation, iron ion homeostasis DNA-binding transcription factor activity regulation of DNA-templated transcription protein dimerization activity, beta-amylase activity
%Germination_15DAS	S7_929082	7	929,082	1.30E-08	8.983	transferase activity, defence response
%Germination_21DAS	S9_15924439	9	15,924,439	4.73E-07	-9.8604	protein glycosylation, transferase activity, DNA binding, cell wall modification, enzyme inhibitor activity, transaminase activity, biosynthetic process, catalytic activity metal ion binding, response to abscisic acid negative regulation of abscisic acid-activated signalling pathway, ethylene-activated signalling pathway, DNA-binding transcription factor activity, regulation of DNA-templated transcription

%Ger15: Percentage of germination in the experiment where water was withdrawn 15 days after sowing; %Ger21: Percentage of germination in the experiment where water was withdrawn 21 days after sowing

### Putative candidate gene identification

The LD decay distance was calculated to identify the putative candidate genes in the vicinity of the SNPs identified in the present study. The decay of LD was 77.217 kb (Fig. 4G). We used the *O. sativa* Nipponbare reference genome (version 7.0) and took the 77.217 kb LD block to define the putative candidate gene search space around the significant SNP marker. We mapped the SNP markers to *O. sativa Nipponbare reference genome* and fetched all the annotated genes in 38.6 kb upstream and 38.6 kb downstream region around the SNP marker (https://plants.ensembl.org/biomart/martview). Further the putative candidate genes reported to be associated with molecular function such as peptidases, proteases, and transferases; polyamine transmembrane transporter activity metabolic processes involving nitrogen compound, response to abiotic/biotic stresses, inflorescence, and reproductive development. Some candidate genes displayed their role in organization of cellular components such as membrane, nucleus, cytoplasm, and ribosome (Table 3).

### Selection of promising breeding lines

To identify stable breeding lines with high grain yield and anaerobic germination, a GGE biplot analysis was conducted. The first two principal components accounted for 94.6% of the total GGE variation (PC1 = 65.8%, PC2 = 28.8%) as shown in Fig. S2. The ranking of breeding lines based on their average grain yield and stability across different years and treatments allowed for the selection of the top 10 promising breeding lines exhibiting both high and stable yield, identified MTAs and the anaerobic germination trait (Table 4).

**Table 4:** The list of selected promising breeding lines with contrasting grain yield (GY; kg ha^-1^) derived from the pooled mean over three years under different treatments

Cross name	Designation	%Germination_ 15DAS	%Germination_ 21DAS	GY	PC1	PC2	MTAs
PR126/IR14D155	Ag-165	85	74	4046	75.24	-23.63	S6_18028538, S6_24492161, S9_15924439
PR126/IR15D120	Ag-399	73	60	4688	60.86	-9.71	S6_18028538, S6_24492161, S9_15924439
PR126/IR15D120	Ag-378	73	60	3809	60.86	-9.71	S6_18028538, S6_24492161, S4_2823374, S7_929082, S9_15924439
PR126/IR10F365	Ag-23	75	74	3814	91.58	4.16	S3_21089181, S6_24492161, S4_2823374, S7_21442267, S9_15924439
PR126/IR 127152-2-10	Ag-230	85	74	3679	91.58	4.16	S4_2823374, S5_14285254, S9_15924439
PR126/IR 127152-2-10	Ag-69	80	60	4264	65.20	-13.91	S4_2823374, S5_14285254, S9_15924439
PR126/IR 127152-2-10	Ag-293	78	60	3981	65.20	-13.91	S3_25375913, S4_2823374, S5_14285254, S9_15924439
PR126/IR 127152-2-10	Ag-404	77	60	3807	60.86	-9.71	S5_14285254, S9_15924439
PR126/IR 127155-1-22	Ag-377	77	65	4870	57.73	-6.68	S3_21089181, S4_2823374, S7_21442267, S9_15924439
PR126/IR 127155-1-22	Ag-2	72	60	4256	57.73	-6.68	S3_21089181, S4_2823374, S7_21442267, S9_15924439
Parents	PR126	9	7	2272	-25.63	-3.75	-
	IR14D155	76	60	2034	66.49	0.39	S3_21089181, S3_25375913, S4_2823374, S5_14285254, S6_18028538, S6_24492161, S9_15924439
	IR15D120	75	65	1774	74.79	-13.58	S3_24412222, S3_25375913, S4_2823374, S5_14285254, S6_18028538, S6_24492161, S7_929082, S9_15924439
	IR13F474	72	65	2028	74.27	-12.47	S3_21089181, S5_14285254, S6_18028538, S6_24492161, S9_15924439
	IR10F365	78	77	1669	95.11	-14.80	S3_21089181, S3_25375913, S4_2823374, S6_18028538, S6_24492161, S9_15924439
	IR 127152-2-10	67	59	1638	64.19	-7.29	S3_24412222, S3_25375913, S4_2823374, S5_14285254, S9_15924439
	IR 127155-1-22	68	65	1567	74.55	-1.92	S3_21089181, S4_2823374, S5_14285254, S7_21442267, S9_15924439
	IR 93312-30-101-20-13-66-6	73	58	1492	62.43	-12.75	S3_25375913, S4_2823374, S5_14285254, S6_18028538, S6_24492161
Environment mean	2021	36	25	2853	0.63	0.66	
	2022	40	18	1166	0.52	-0.75	
	2023	38	22	2499	0.58	-0.05	

%Ger15: Percentage of germination in the experiment where water was withdrawn 15 days after sowing; %Ger21: Percentage of germination in the experiment where water was withdrawn 21 days after sowing, GY: grain yield (kg/ha), PC1: Principal component 1, PC2: Principal component 2, MTAs: marker-trait associations

## Discussion

Anaerobic germination trait is significant in agriculture, especially in the cultivation of rice, where submerged conditions prevail just after sowing. Understanding the mechanisms behind anaerobic germination is crucial for optimizing crop yields in flooded fields, as well as for studying the evolutionary adaptations of plants to direct seeded environmental conditions. Significant variations were observed among the traits in the present study, indicating substantial genetic diversity within the population. These variations provide opportunities for selecting desirable traits, particularly for improving anaerobic germination and grain yield. The high heritability highlights strong genetic control of the trait under anaerobic conditions, with consistency across years reinforcing its stability for selection in breeding. The gradual increase in germination percentages indicates potential genetic gains through targeted selection, despite environmental variability.

The advanced molecular biology techniques, such as genome sequencing, quantitative trait locus (QTL) mapping, and association mapping can pinpoint regions of the genome that are correlated with traits related to anaerobic germination, such as seed vigor, tolerance to submergence, and efficient utilization of stored energy reserves. The mapping of genomic regions for anaerobic germination under direct seeded cultivation conditions represents a promising approach to advancing crop improvement efforts and ensuring food security in the face of changing environmental conditions and agricultural practices. The present study represents genomic characterization of the anaerobic germination using 384 breeding lines derived from nested association mapping population using genome-wide association mapping approach. Association mapping has been utilized in rice to identify new genetic regions linked to various complex traits, such as anaerobic germination [13, 16, 17, 24], grain yield [25–27], grain quality [28, 29], blast resistance [30], and responses to environmental stress [31, 32].

For the anaerobic germination trait, 11 MTAs have been identified across six rice chromosomes: 3, 4, 5, 6, and 7. Previous studies have found QTLs/MTAs on chromosomes 1, 2, 3, 5, 7, 9, and 11 [8, 10, 33, 34]. Notably, the MTAs from the current study overlap with the QTL regions reported by Angaji et al. [10, 33]. Additionally, two SNPs associated with anaerobic germination, K9_12253431 and K9_12253887, were identified on chromosome 9 [35]. In this study, the MTA identified on chromosome 1 coincided with a previously reported QTL for days to 50% flowering under direct-seeded cultivation conditions [36]. Interestingly, the MTA S10_17012689 identified on chromosome 10 in this study was in the QTL region *qGY10.1*, which is associated with grain yield under direct-seeded cultivation conditions as reported by Sandhu et al. [37]. Similarly, the MTAs identified on chromosomes 1, 2, and 8 in this study coincided with previously reported QTLs. These include *qDTY*_*1.1*_ for grain yield under reproductive stage drought stress conditions [38] and *qGY1.1* under direct-seeded cultivation conditions on chromosome 1 [35]. Additionally, *qGY2.1* and *qGY2.2* on chromosome 2, and *qGY8.1* on chromosome 8 were identified for grain yield under direct-seeded cultivation conditions [37].

The average LD decay value was estimated at 77.217 kb, where LD decays to half of its maximum value. The putative candidate genes identified in the genomic regions underlying MTAs are associated with molecular functions such as peptidases, proteases, and transferases; polyamine transmembrane transporter activity; nitrogen compound metabolic processes; response to abiotic and biotic stresses; and inflorescence and reproductive development. Some candidate genes are involved in the organization of cellular components such as the membrane, nucleus, cytoplasm, and ribosome. The application of exogenous polyamines (PAs) has been shown to enhance plant tolerance to various abiotic stresses, including salinity, drought, waterlogging, osmotic stress, heavy metals, and extreme temperatures [39–43]. Peptidases and proteases break down proteins into peptides and amino acids, providing nutrients and aiding in cellular repair and stress responses during anaerobic conditions. Transferases facilitate the transfer of functional groups between molecules, vital for metabolic processes and energy production, helping plants cope with oxygen deficiency. Efficient nitrogen compound metabolism is essential for synthesizing amino acids and nucleotides, supporting growth and development even in low-oxygen environments. Together, these functions may enhance the resilience and adaptive capacity of plants during anaerobic germination. The top 10 selected breeding lines, which exhibit stable yield and enhanced germination under anaerobic conditions, could serve as valuable new breeding material for genomics-assisted introgression programs.

Through the selection of promising breeding lines carrying specific alleles linked to improved anaerobic germination, breeders can create varieties better suited for direct-seeded cultivation conditions. This approach aims to increase yields and enhance resilience against environmental stresses.

## Conclusions

The present study successfully standardized the phenotyping method for anaerobic germination. The results demonstrated that the phenotyping methods using plastic cups, plug trays with field soil, and plastic trays were comparable to natural field conditions. Characterization of the phenotypic traits revealed significant variations in germination among the breeding lines, with positive checks beginning to germinate from the seventh day under anaerobic conditions. GWAS analysis revealed 19 marker-trait associations (MTAs) for anaerobic germination, flowering time, and grain yield. Notably, significant SNPs associated with germination were detected on chromosomes 3, 4, 5, 6, 7, and 9. For flowering and yield traits, significant SNPs were identified on chromosomes 1, 2, 5, 8, and 10. Putative candidate genes within the LD decay distance of significant SNPs were identified, suggesting roles in various molecular functions and stress responses. Finally, GGE biplot analysis highlighted the top 10 promising breeding lines with high and stable yields and anaerobic germination traits, marking them as suitable candidates for future breeding programs.

## Materials and methods

The experimental plant material comprised of nested association mapping population of 384 genotypes derived from seven different donor parents (IR14D155, IR15D120, IR13F474, IR10F365, IR 127152-2-10, IR 127155-1-22, IR 93312-30-101-20-13-66-6) for anaerobic germination and the cultivated variety of Punjab was used as a recipient parent (PR126). The phenotypic characterization was carried out in the field and screenhouse areas of School of Agricultural Biotechnology, Punjab Agricultural University, Ludhiana, India. The seven donors along with checks (PR121, PR126, PR128, PR129, PB1509) were screened first for germination under anaerobic conditions in plastic trays in 2018. The donors were used to develop the bi-parental mapping population in the background of PR126 in 2018–2019. A total of 157 to 274 F_1_s seeds were produced. The F_1_ seeds were advanced to F_2_ in 2019–2020 rabi season under controlled conditions in screenhouse. The 1429 F_2_s plants were advanced to F_3_ and F_4_ under field conditions in 2020 *Kharif* season. The 384 F_4_ breeding lines were randomly chosen and the breeding lines at F_4_, F_5_ and F_6_ generations were evaluated under screenhouse and field conditions in 2021, 2022 and 2023 *kharif* seasons, respectively. The details on the 384 breeding lines from seven different crosses are presented in Fig. 1.

### Standardization of phenotypic screening for anaerobic germination

Seven distinct experiments were conducted to simulate anaerobic germination, specifically: (i) anaerobic germination with water in plastic cups, (ii) anaerobic germination with soil and water in plastic cups, (iii) anaerobic germination with water and germination paper in plastic cups, (iv) anaerobic germination in plug trays filled with field soil placed on a concrete screening table, (v) anaerobic germination in plug trays placed in plastic trays (each measuring 60 cm in length, 40 cm in width, and 15 cm in height), (vi) anaerobic germination directly on the puddled soil of a screenhouse to provide more natural field conditions, and (vii) anaerobic germination under natural field conditions (Fig. 2). These experiments aimed to standardize phenotypic screening for anaerobic germination. Each experiment included one local check variety sensitive to anaerobic conditions (PR126) and five different donors (IR14D155, IR13F474, IR10F365, IR127155-1-22, and IR93312-30-101-20-13-66-6). Control experiments were maintained for each treatment. The experiments took place during the kharif season of 2020 at the screenhouse and field area of the School of Agricultural Biotechnology, PAU, Ludhiana. Dry seeds were used, with dormancy broken by keeping the seeds at 50 °C for 3 days in a hot-air oven. A completely randomized design with three replications was implemented for all conditions.

In each experiment, fourteen seeds from each entry were sown, and the lines were covered with fine field soil. The trays were carefully submerged to maintain 15 cm of water above the soil surface throughout the experiment (15 days). The experiments were continuously monitored for percent germination and water level maintenance. The number of seedlings that emerged above the water surface was counted on the 21st day.

### Phenotypic screening for anaerobic germination

The nested association mapping populations derived from the crossing of seven donors with PR126 were screened for germination under anaerobic conditions using Completely Randomized Design in two replications in 2021, 2022 and 2023 *kharif* seasons. Three different experiments were carried out in each year.

- In the first experiment, a total of seven desiccated seeds from each breeding line were planted at a soil depth of 2–3 cm in a linear arrangement, with 14 lines per plug tray. In each tray, one of the donor parents was used as positive control and PR126 was used as negative control. The water level was maintained at 15 cm above the plug tray surface for 15 days. The water was removed after 15 days and the seeds were allowed to germinate for the next 10 days. The observations on the percent germination were recorded from the fifth day after germination till 20th day. The phenotypic data was collected by counting of germinated plants per breeding line.
- In other set of experiment, a total of seven desiccated seeds from each breeding line were planted at a soil depth of 2–3 cm in a linear arrangement, with 14 lines per plug tray. In each tray, one of the donor parents was used as positive control and PR126 was used as negative control. The water level was maintained at 15 cm above the tray surface for 21 days. After 21 days, the water was removed, the seeds were allowed to germinate for the next 10 days. The observations on the percent germination were recorded from the fifth day after germination till 26th day.
- A control group of treatments was also maintained by adequately hydrating the soil surface. In the control experimental group, all the mapping population lines were uniformly seeded in trays and were not subjected to standing water following the sowing.

### Phenotypic evaluation of breeding lines under field conditions

The phenotypic characterization of the 384 breeding lines was carried out under field conditions in 2021, 2022 and 2023 *kharif* seasons, respectively. The breeding lines were evaluated under anaerobic conditions in field as well in 2021. These lines were planted in an augmented design in 2021 and alpha lattice design in 2022 and 2023, kharif seasons in the field. The cultivation involved direct-seeded planting, with the breeding lines being grown in paired row plots measuring each 1.5 m in size. The spacing between rows and between individual plants was maintained at 20 cm each and a control group of treatments was also maintained by adequately hydrating the soil surface for the anaerobic germination experiment. The field study involved the collection of phenotypic data pertaining to days to 50% flowering (DTF), plant height (PHT), number of panicles per plant (P/P), panicle length (PL), and grain yield (GY) for all the experiments across years. The data on %germination was collected for the experiment involving screening of lines under anaerobic conditions.

The DTF was recorded when half of the plants in the paired row plot had initiated their panicles. The PHT on three random plants was measured from the base of the plant to the tip of the main panicle at the level of maturity. The PL was measured using a centimeter scale and the P/P was counted manually counting the number of panicles per plant. Upon reaching physiological maturity, the grains were harvested, threshed, dried, and weighted (g). The GY in kg ha^− 1^ was calculated.

### Statistical analysis

Means were calculated from replicated observations. Means were used to draw frequency curves to know the phenotypic distribution of the traits. The analysis of variance (ANOVA) across experiments and years was calculated in PBTools V 1.4.0. The Fisher’s t-test was used to determine the significant difference across years, and breeding lines and to estimate the interactions. The correlation analysis among traits was performed in R. v.1.1.423.

The phenotypic stability and grain yield adaptability of the breeding lines across years were evaluated using the GGE biplot analysis, considering the effects of genotype (G) and genotype by environment (GE) as random. The best linear unbiased prediction (BLUP) values of the G and GE effects were calculated. The multiplicative model in PB tool version 1.3 (bbi.irri.orgbbi.irri.org) was used to explain the relationship between breeding lines and years. To assess the phenotypic stability and grain yield adaptability of the breeding lines across different seasons and treatments, GGE biplot analysis was conducted, treating genotype (G) and genotype-by-environment (GE) effects as random. The best linear unbiased prediction (BLUP) values for the G and GE effects were calculated. The multiplicative model in PB tool version 1.3 (bbi.irri.org) was employed to elucidate the relationship between genotype and seasons.

### Genotypic characterization

Genomic DNA of the nested association F_4_ mapping population, including donor and recipient parents extracted from 21 days old seedlings. The quantification and quality of the isolated DNA were done using gel electrophoresis and Nanodrop 1000 spectrophotometer and then subjected to high throughput ddRAD sequencing using Illumina HiSeq 4000. The dual enzyme (ddRAD) digested libraries were prepared and run on a bioanalyzer for the library profiling. The raw sequences were generated. The paired-end sequencing and the read processing were carried out at NGB diagnostics Private Limited, New Delhi (India). First, the Illumina adaptor sequences were removed and quality trimming of the adaptor-clipped reads was performed. The reads with a final length of 20 or less were rejected from further analysis. The mapping of sequencing reads against the reference genome sequence of *O. sativa* v7.0 (http://rice.plantbiology.msu.edu/pub/data/EukaryoticProjects/osativa/annotationdbs/pseudomolecule/version_7.0/all.dir/*)* using bwa (Burrow-Wheeler Aligner) (version 0.7.17-r1188) was performed. The read pairs with both read aligning in the expected orientation were used for further analyses. For the conversion of mapping files from the sam alignment format to the bam binary format, SAMtools version 0.1.19 [44] was used. Picard software (version 1.48) was then used to sort the bam files and remove any PCR duplicates. Finally, bam files were processed using the unified genotyper of Genome Analysis Toolkit, version (GATK pipeline) for the variant calling. The Vcftools (version 0.1.17) was used for the filtering of variant calls (Supplementary information). Finally, the samples with minor allele frequency (MAF) 5% and missing 10% were kept.

### Genome wide association study (GWAS)

The genotyping of the nested association F_4_ mapping population constituting 384 breeding lines was conducted using ddRADseq (Double Digest Random Amplified DNA associated Sequencing). The ddRADseq analysis was conducted by NGB Private Ltd., located in New Delhi. The raw files were processed and the hapmap file containing SNP data was generated. The genotypic data of the SNP was subjected to a filtering process, wherein any SNP with missing data above 10% and a minor allele frequency (MAF) below 0.05 was excluded. The Genome-Wide Association Study (GWAS) was performed using the “FarmCPU” method. This analysis was conducted using the GAPIT (Genomic Association and Prediction Integrated Tool) package within the R statistical environment (R Core Team, 2018). The primary objective of this analysis was to compute the predicted p-values for each single nucleotide polymorphism (SNP). FarmCPU method based on the Mixed Linear Model (MLM) framework considers both the population structure and the kinship to reduce the number of false positive results. The estimation of population structure was conducted using principal component analysis (PCA) in GAPIT version 3.0 package [45] as well as using the ADMIXTURE v1.3.0 [46]. The p-values associated with each single nucleotide polymorphism (SNP) were visualized on Manhattan plots, where the log(p) values were used for plotting.

### Postulation of candidate genes

The pairwise linkage disequilibrium between the SNP loci at the chromosome level with no missing data was calculated in Tassel v 5.0 and plotted by computing the r^2^ estimators between all pairs of SNP markers using the R. The 95th percentile of the r^2^ values was used as the estimator of short-range LD, and the distance at which the short-range LD was halved was used as the estimator of LD distance. The chromosome-wise linkage disequilibrium (LD) decay value (in Mb) was used to define the confidence intervals to locate candidate genes. The mapped SNPs linked to significant genomic regions were mined for potential candidate genes. The putative candidate gene underlying the MTAs were surveyed considering LD decay using Ensemble plant (http://plants.ensembl.org/index.html) database.

## Electronic supplementary material

Below is the link to the electronic supplementary material.

Supplementary Material 1

## Data Availability

The ddRADseq data that support the findings of this study have been deposited in the NCBI under BioProject ID PRJNA1161833. All data generated or analysed during this study are included in this manuscript.
