# Supplementary material for: Understanding anaerobic germination in direct-seeded rice: a genomic mapping approach
Source: BMC Plant Biol. 2024 Dec 19;24:1194. doi: 10.1186/s12870-024-05901-z (PMC11656897; doi:10.1186/s12870-024-05901-z)
Supplement: Supplementary file 1 — Supplementary Material 1 [file 12870_2024_5901_MOESM1_ESM.docx]

**Table S1. Detailed information on the codes mentioned in the Fig. S2**

| **Numeric code** | **Designation** | **Cross name** | **Grain yield (Kg/ha)** | **Percent germination where the water was removed after 15 days** | **Percent germination where the water was removed after 21 days** |
| --- | --- | --- | --- | --- | --- |
| 1 | Ag-215 | PR126/IR 127152-2-10 | 3101 | 41.7 | 0.0 |
| 2 | Ag-403 | PR126/IR 127152-2-10 | 1318 | 20.3 | 20.6 |
| 3 | Ag-278 | PR126/IR 127152-2-10 | 1145 | 64.0 | 40.2 |
| 4 | Ag-284 | PR126/IR 127152-2-10 | 2845 | 64.0 | 12.5 |
| 5 | Ag-332 | PR126/IR 127152-2-10 | 2973 | 21.6 | 12.5 |
| 6 | Ag-143 | PR126/IR 127152-2-10 | 1890 | 27.4 | 6.3 |
| 7 | Ag-81 | PR126/IR 127152-2-10 | 1380 | 37.3 | 27.7 |
| 8 | Ag-488 | PR126/IR 127152-2-10 | 2117 | 15.2 | 0.0 |
| 9 | Ag-139 | PR126/IR 127152-2-10 | 1731 | 41.7 | 13.4 |
| 10 | Ag-248 | PR126/IR 127152-2-10 | 1638 | 50.1 | 0.0 |
| 11 | Ag-425 | PR126/IR 127152-2-10 | 2653 | 83.8 | 48.2 |
| 12 | Ag-404 | PR126/IR 127152-2-10 | 3807 | 77.4 | 57.1 |
| 13 | Ag-297 | PR126/IR 127152-2-10 | 2534 | 35.5 | 12.5 |
| 14 | Ag-308 | PR126/IR 127152-2-10 | 3517 | 42.7 | 26.8 |
| 15 | Ag-336 | PR126/IR 127152-2-10 | 4010 | 35.9 | 19.7 |
| 16 | Ag-141 | PR126/IR 127152-2-10 | 2167 | 48.8 | 7.2 |
| 17 | Ag-315 | PR126/IR 127152-2-10 | 2753 | 21.2 | 19.7 |
| 18 | Ag-208 | PR126/IR 127152-2-10 | 1521 | 34.6 | 7.2 |
| 19 | Ag-330 | PR126/IR 127152-2-10 | 2276 | 64.4 | 26.8 |
| 20 | Ag-210 | PR126/IR 127152-2-10 | 1960 | 56.0 | 26.8 |
| 21 | Ag-373 | PR126/IR 127152-2-10 | 2131 | 24.0 | 26.8 |
| 22 | Ag-63 | PR126/IR 127152-2-10 | 1992 | 51.4 | 34.0 |
| 23 | Ag-175 | PR126/IR 127152-2-10 | 1466 | 34.6 | 7.2 |
| 24 | Ag-328 | PR126/IR 127152-2-10 | 2200 | 42.7 | 26.8 |
| 25 | Ag-155 | PR126/IR 127152-2-10 | 3945 | 91.7 | 57.1 |
| 26 | Ag-99 | PR126/IR 127152-2-10 | 2477 | 65.8 | 19.7 |
| 27 | Ag-240 | PR126/IR 127152-2-10 | 1936 | 64.4 | 26.8 |
| 28 | Ag-89 | PR126/IR 127152-2-10 | 1102 | 44.5 | 40.2 |
| 29 | Ag-8 | PR126/IR 127152-2-10 | 1792 | 0.0 | 0.0 |
| 30 | Ag-78 | PR126/IR 127152-2-10 | 1572 | 51.4 | 41.1 |
| 31 | Ag-234 | PR126/IR 127152-2-10 | 2341 | 78.7 | 40.2 |
| 32 | Ag-140 | PR126/IR 127152-2-10 | 1989 | 27.4 | 13.4 |
| 33 | Ag-326 | PR126/IR 127152-2-10 | 3465 | 0.0 | 0.0 |
| 34 | Ag-231 | PR126/IR 127152-2-10 | 646 | 7.2 | 0.0 |
| 35 | Ag-261 | PR126/IR 127152-2-10 | 1997 | 50.1 | 19.7 |
| 36 | Ag-249 | PR126/IR 127152-2-10 | 1894 | 78.7 | 34.0 |
| 37 | Ag-346 | PR126/IR 127152-2-10 | 1732 | 43.0 | 0.0 |
| 38 | Ag-316 | PR126/IR 127152-2-10 | 2248 | 0.0 | 0.0 |
| 39 | Ag-130 | PR126/IR 127152-2-10 | 2198 | 48.8 | 26.8 |
| 40 | Ag-353 | PR126/IR 127152-2-10 | 2451 | 35.8 | 26.8 |
| 41 | Ag-402 | PR126/IR 127152-2-10 | 3691 | 77.4 | 71.4 |
| 42 | Ag-474 | PR126/IR 127152-2-10 | 3434 | 0.9 | 0.0 |
| 43 | Ag-131 | PR126/IR 127152-2-10 | 1503 | 56.0 | 20.6 |
| 44 | Ag-398 | PR126/IR 127152-2-10 | 3560 | 6.0 | 0.0 |
| 45 | Ag-170 | PR126/IR 127152-2-10 | 2293 | 41.7 | 34.8 |
| 46 | Ag-412 | PR126/IR 127152-2-10 | 3185 | 63.1 | 28.6 |
| 47 | Ag-166 | PR126/IR 127152-2-10 | 1291 | 20.3 | 27.7 |
| 48 | Ag-88 | PR126/IR 127152-2-10 | 2033 | 58.7 | 41.1 |
| 49 | Ag-266 | PR126/IR 127152-2-10 | 1656 | 14.0 | 6.3 |
| 50 | Ag-461 | PR126/IR 127152-2-10 | 1162 | 72.3 | 13.4 |
| 51 | Ag-123 | PR126/IR 127152-2-10 | 3323 | 70.2 | 45.6 |
| 52 | Ag-74 | PR126/IR 127152-2-10 | 2828 | 44.2 | 47.3 |
| 53 | Ag-305 | PR126/IR 127152-2-10 | 2823 | 49.8 | 33.1 |
| 54 | Ag-115 | PR126/IR 127152-2-10 | 1397 | 51.6 | 59.8 |
| 55 | Ag-109 | PR126/IR 127152-2-10 | 1430 | 1.6 | 0.0 |
| 56 | Ag-190 | PR126/IR 127152-2-10 | 1852 | 0.0 | 7.2 |
| 57 | Ag-5 | PR126/IR 127152-2-10 | 1459 | 85.7 | 62.5 |
| 58 | Ag-394 | PR126/IR 127152-2-10 | 2040 | 13.2 | 0.0 |
| 59 | Ag-156 | PR126/IR 127152-2-10 | 1520 | 48.8 | 26.8 |
| 60 | Ag-69 | PR126/IR 127152-2-10 | 4264 | 79.9 | 59.8 |
| 61 | Ag-409 | PR126/IR 127152-2-10 | 1541 | 48.9 | 20.6 |
| 62 | Ag-185 | PR126/IR 127152-2-10 | 1921 | 63.1 | 26.8 |
| 63 | Ag-113 | PR126/IR 127152-2-10 | 3672 | 80.1 | 59.8 |
| 64 | Ag-169 | PR126/IR 127152-2-10 | 2263 | 27.5 | 14.3 |
| 65 | Ag-260 | PR126/IR 127152-2-10 | 2411 | 14.4 | 0.0 |
| 66 | Ag-36 | PR126/IR 127152-2-10 | 1498 | 21.9 | 13.4 |
| 67 | Ag-376 | PR126/IR 127152-2-10 | 3209 | 88.3 | 60.7 |
| 68 | Ag-30 | PR126/IR 127152-2-10 | 1792 | 64.7 | 40.2 |
| 69 | Ag-369 | PR126/IR 127152-2-10 | 1006 | 45.4 | 7.2 |
| 70 | Ag-352 | PR126/IR 127152-2-10 | 2573 | 50.1 | 26.8 |
| 71 | Ag-471 | PR126/IR 127152-2-10 | 2792 | 22.4 | 27.7 |
| 72 | Ag-216 | PR126/IR 127152-2-10 | 2243 | 48.9 | 12.5 |
| 73 | Ag-333 | PR126/IR 127152-2-10 | 1687 | 21.6 | 6.3 |
| 74 | Ag-1 | PR126/IR 127152-2-10 | 1603 | 0.0 | 0.0 |
| 75 | Ag-87 | PR126/IR 127152-2-10 | 1997 | 30.2 | 33.1 |
| 76 | Ag-410 | PR126/IR 127152-2-10 | 2049 | 34.6 | 20.6 |
| 77 | Ag-384 | PR126/IR 127152-2-10 | 3534 | 74.0 | 19.7 |
| 78 | Ag-439 | PR126/IR 127152-2-10 | 1831 | 12.4 | 0.0 |
| 79 | Ag-429 | PR126/IR 127152-2-10 | 3486 | 76.6 | 64.3 |
| 80 | Ag-392 | PR126/IR 127152-2-10 | 2715 | 31.2 | 20.6 |
| 81 | Ag-182 | PR126/IR 127152-2-10 | 2177 | 48.9 | 33.1 |
| 82 | Ag-275 | PR126/IR 127152-2-10 | 2137 | 28.3 | 33.1 |
| 83 | Ag-219 | PR126/IR 127152-2-10 | 2448 | 56.0 | 20.6 |
| 84 | Ag-79 | PR126/IR 127152-2-10 | 1556 | 44.2 | 27.7 |
| 85 | Ag-256 | PR126/IR 127152-2-10 | 2566 | 57.3 | 33.1 |
| 86 | Ag-14 | PR126/IR 127152-2-10 | 2981 | 85.7 | 74.1 |
| 87 | Ag-173 | PR126/IR 127152-2-10 | 1868 | 13.2 | 7.2 |
| 88 | Ag-416 | PR126/IR 127152-2-10 | 3157 | 0.0 | 0.0 |
| 89 | Ag-415 | PR126/IR 127152-2-10 | 1952 | 34.6 | 14.3 |
| 90 | Ag-290 | PR126/IR 127152-2-10 | 2481 | 49.7 | 27.7 |
| 91 | Ag-300 | PR126/IR 127152-2-10 | 1334 | 49.8 | 7.2 |
| 92 | Ag-324 | PR126/IR 127152-2-10 | 2067 | 42.6 | 26.8 |
| 93 | Ag-18 | PR126/IR 127152-2-10 | 1589 | 7.6 | 7.2 |
| 94 | Ag-317 | PR126/IR 127152-2-10 | 2553 | 0.0 | 0.0 |
| 95 | Ag-111 | PR126/IR 127152-2-10 | 2522 | 58.7 | 53.6 |
| 96 | Ag-361 | PR126/IR 127152-2-10 | 1995 | 31.2 | 7.2 |
| 97 | Ag-226 | PR126/IR 127152-2-10 | 2557 | 48.9 | 20.6 |
| 98 | Ag-122 | PR126/IR 127152-2-10 | 2259 | 34.6 | 20.6 |
| 99 | Ag-388 | PR126/IR 127152-2-10 | 1715 | 38.3 | 27.7 |
| 100 | Ag-357 | PR126/IR 127152-2-10 | 3371 | 43.0 | 33.1 |
| 101 | Ag-445 | PR126/IR 127152-2-10 | 792 | 33.8 | 26.8 |
| 102 | Ag-58 | PR126/IR 127152-2-10 | 2031 | 65.6 | 27.7 |
| 103 | Ag-199 | PR126/IR 127152-2-10 | 2107 | 41.7 | 7.2 |
| 104 | Ag-285 | PR126/IR 127152-2-10 | 2495 | 56.9 | 41.1 |
| 105 | Ag-101 | PR126/IR 127152-2-10 | 3716 | 51.6 | 12.5 |
| 106 | Ag-92 | PR126/IR 127152-2-10 | 1897 | 65.9 | 42.9 |
| 107 | Ag-356 | PR126/IR 127152-2-10 | 2060 | 14.3 | 0.0 |
| 108 | Ag-41 | PR126/IR 127152-2-10 | 1359 | 51.4 | 26.8 |
| 109 | Ag-21 | PR126/IR 127152-2-10 | 2239 | 79.0 | 74.1 |
| 110 | Ag-262 | PR126/IR 127152-2-10 | 580 | 14.4 | 13.4 |
| 111 | Ag-401 | PR126/IR 127152-2-10 | 2041 | 20.3 | 21.5 |
| 112 | Ag-205 | PR126/IR 127152-2-10 | 2904 | 41.7 | 7.2 |
| 113 | Ag-273 | PR126/IR 127152-2-10 | 3442 | 85.4 | 59.8 |
| 114 | Ag-466 | PR126/IR 127152-2-10 | 2273 | 15.2 | 20.6 |
| 115 | Ag-129 | PR126/IR 127152-2-10 | 2070 | 63.1 | 25.9 |
| 116 | Ag-413 | PR126/IR 127152-2-10 | 1599 | 84.6 | 28.6 |
| 117 | Ag-340 | PR126/IR 127152-2-10 | 2768 | 35.9 | 19.7 |
| 118 | Ag-126 | PR126/IR 127152-2-10 | 2395 | 56.0 | 60.7 |
| 119 | Ag-477 | PR126/IR 127152-2-10 | 2056 | 22.4 | 13.4 |
| 120 | Ag-110 | PR126/IR 127152-2-10 | 1683 | 44.4 | 41.1 |
| 121 | Ag-162 | PR126/IR 127152-2-10 | 619 | 20.3 | 19.7 |
| 122 | Ag-142 | PR126/IR 127152-2-10 | 2562 | 48.8 | 26.8 |
| 123 | Ag-379 | PR126/IR 127152-2-10 | 2195 | 9.7 | 7.2 |
| 124 | Ag-453 | PR126/IR 127152-2-10 | 3236 | 62.3 | 25.9 |
| 125 | Ag-414 | PR126/IR 127152-2-10 | 2188 | 41.8 | 27.7 |
| 126 | Ag-291 | PR126/IR 127152-2-10 | 1526 | 56.8 | 13.4 |
| 127 | Ag-186 | PR126/IR 127152-2-10 | 1468 | 34.6 | 20.6 |
| 128 | Ag-20 | PR126/IR 127152-2-10 | 2345 | 71.9 | 45.6 |
| 129 | Ag-133 | PR126/IR 127152-2-10 | 1540 | 77.4 | 0.0 |
| 130 | Ag-196 | PR121 | 1796 | 0.0 | 0.0 |
| 131 | Ag-71 | PR126/IR 127152-2-10 | 3983 | 44.2 | 41.1 |
| 132 | Ag-54 | PR126/IR 127152-2-10 | 1706 | 72.8 | 34.8 |
| 133 | Ag-52 | PR126/IR 127152-2-10 | 2720 | 37.1 | 34.0 |
| 134 | Ag-145 | PR126/IR 127152-2-10 | 1753 | 63.1 | 7.2 |
| 135 | Ag-485 | PR126/IR 127152-2-10 | 3859 | 65.2 | 41.1 |
| 136 | Ag-463 | PR126/IR 127152-2-10 | 3059 | 65.2 | 21.5 |
| 137 | Ag-200 | PR126/IR 127152-2-10 | 1482 | 63.1 | 0.0 |
| 138 | Ag-94 | PR126/IR 127152-2-10 | 3169 | 23.0 | 21.5 |
| 139 | Ag-221 | PR126/IR 127152-2-10 | 1946 | 41.8 | 7.2 |
| 140 | Ag-338 | PR126/IR 127152-2-10 | 1540 | 57.2 | 52.7 |
| 141 | Ag-188 | PR126/IR 127152-2-10 | 974 | 0.0 | 28.6 |
| 142 | Ag-313 | PR126/IR 127152-2-10 | 2301 | 21.2 | 53.6 |
| 143 | Ag-442 | PR126/IR 127152-2-10 | 1992 | 12.4 | 20.6 |
| 144 | Ag-293 | PR126/IR 127152-2-10 | 3981 | 78.3 | 59.8 |
| 145 | Ag-67 | PR126/IR 127152-2-10 | 1864 | 30.0 | 0.0 |
| 146 | Ag-411 | PR126/IR 127152-2-10 | 1743 | 56.0 | 26.8 |
| 147 | Ag-135 | PR126/IR 127152-2-10 | 1684 | 13.1 | 0.0 |
| 148 | Ag-230 | PR126/IR 127152-2-10 | 3679 | 84.6 | 74.1 |
| 149 | Ag-59 | PR126/IR 127152-2-10 | 1745 | 51.4 | 20.6 |
| 150 | Ag-95 | PR126/IR 127152-2-10 | 3777 | 51.6 | 19.7 |
| 151 | Ag-483 | PR126/IR 127152-2-10 | 2642 | 50.9 | 13.4 |
| 152 | Ag-436 | PR126/IR 127152-2-10 | 2372 | 26.6 | 21.5 |
| 153 | Ag-11 | PR126/IR 127152-2-10 | 2401 | 64.7 | 34.8 |
| 154 | Ag-254 | PR126/IR 127152-2-10 | 2122 | 43.0 | 0.0 |
| 155 | Ag-272 | PR126/IR 127152-2-10 | 926 | 49.7 | 34.8 |
| 156 | Ag-172 | PR126/IR 127152-2-10 | 1277 | 20.3 | 0.0 |
| 157 | Ag-252 | PR126/IR 127152-2-10 | 1613 | 71.5 | 53.6 |
| 158 | Ag-259 | PR126/IR 127152-2-10 | 2422 | 43.0 | 20.6 |
| 159 | Ag-364 | PR126/IR 127152-2-10 | 984 | 45.4 | 0.0 |
| 160 | Ag-84 | PR126/IR 127152-2-10 | 2494 | 37.3 | 54.5 |
| 161 | Ag-283 | PR126/IR 127152-2-10 | 1856 | 56.8 | 26.8 |
| 162 | Ag-4 | PR126/IR 127152-2-10 | 3539 | 100.0 | 81.3 |
| 163 | Ag-360 | PR126/IR 127152-2-10 | 1537 | 21.6 | 7.2 |
| 164 | Ag-3 | PR126/IR 127152-2-10 | 2228 | 57.6 | 67.0 |
| 165 | Ag-435 | PR126/IR 127152-2-10 | 3400 | 40.9 | 20.6 |
| 166 | Ag-72 | PR126/IR 127152-2-10 | 2000 | 65.6 | 47.3 |
| 167 | Ag-32 | PR126/IR 127152-2-10 | 2814 | 43.4 | 39.3 |
| 168 | Ag-472 | PR126/IR 127152-2-10 | 2217 | 22.4 | 27.7 |
| 169 | MTU1010 | Check | 991 | 54.0 | 42.9 |
| 170 | Ag-255 | PR126/IR14D155 | 1416 | 14.4 | 14.3 |
| 171 | Ag-165 | PR126/IR14D155 | 4046 | 84.6 | 66.1 |
| 172 | Ag-270 | PR126/IR14D155 | 1673 | 49.7 | 19.7 |
| 173 | Ag-418 | PR126/IR14D155 | 1054 | 41.7 | 20.6 |
| 174 | Ag-60 | PR126/IR14D155 | 1586 | 87.1 | 54.5 |
| 175 | Ag-225 | PR126/IR14D155 | 2948 | 41.7 | 39.3 |
| 176 | Ag-114 | PR126/IR14D155 | 2213 | 58.7 | 33.1 |
| 177 | Ag-189 | PR126/IR14D155 | 1377 | 77.4 | 47.3 |
| 178 | Ag-124 | PR126/IR14D155 | 1264 | 56.0 | 33.1 |
| 179 | Ag-433 | PR126/IR14D155 | 1578 | 26.6 | 14.3 |
| 180 | Ag-434 | PR126/IR14D155 | 2222 | 12.4 | 14.3 |
| 181 | Ag-28 | PR126/IR14D155 | 3314 | 93.3 | 74.1 |
| 182 | Ag-100 | PR126/IR14D155 | 1047 | 44.4 | 13.4 |
| 183 | Ag-380 | PR126/IR14D155 | 2115 | 66.8 | 27.7 |
| 184 | Ag-458 | PR126/IR14D155 | 3020 | 0.9 | 0.0 |
| 185 | Ag-444 | PR126/IR14D155 | 1886 | 19.5 | 7.2 |
| 186 | Ag-288 | PR126/IR14D155 | 1631 | 64.0 | 20.6 |
| 187 | Ag-10 | PR126/IR14D155 | 2426 | 43.3 | 55.4 |
| 188 | Ag-424 | PR126/IR14D155 | 1751 | 0.0 | 0.0 |
| 189 | Ag-83 | PR126/IR14D155 | 1777 | 65.8 | 34.0 |
| 190 | Ag-90 | PR126/IR14D155 | 2085 | 37.3 | 34.0 |
| 191 | Ag-368 | PR126/IR14D155 | 2348 | 2.6 | 21.5 |
| 192 | Ag-430 | PR126/IR14D155 | 1859 | 26.6 | 27.7 |
| 193 | Ag-375 | PR126/IR14D155 | 3009 | 2.6 | 21.5 |
| 194 | Ag-467 | PR126/IR14D155 | 1339 | 29.5 | 0.0 |
| 195 | Ag-43 | PR126/IR15D120 | 3109 | 1.4 | 0.0 |
| 196 | Ag-479 | PR126/IR15D120 | 3239 | 0.9 | 0.0 |
| 197 | Ag-222 | PR126/IR15D120 | 1101 | 20.3 | 13.4 |
| 198 | Ag-118 | PR126/IR15D120 | 1575 | 1.6 | 0.0 |
| 199 | Ag-80 | PR126/IR15D120 | 1759 | 44.2 | 19.7 |
| 200 | Ag-13 | PR126/IR15D120 | 2078 | 43.3 | 8.4 |
| 201 | Ag-77 | PR126/IR15D120 | 1653 | 65.6 | 13.4 |
| 202 | Ag-302 | PR126/IR15D120 | 1098 | 7.2 | 0.0 |
| 203 | Ag-47 | PR126/IR15D120 | 1167 | 51.4 | 7.2 |
| 204 | Ag-25 | PR126/IR15D120 | 1742 | 29.1 | 20.6 |
| 205 | Ag-229 | PR126/IR15D120 | 1562 | 84.6 | 41.1 |
| 206 | Ag-9 | PR126/IR15D120 | 2039 | 14.8 | 13.4 |
| 207 | Ag-85 | PR126/IR15D120 | 2138 | 1.6 | 0.0 |
| 208 | Ag-382 | PR126/IR15D120 | 3143 | 16.9 | 7.2 |
| 209 | Ag-318 | PR126/IR15D120 | 2314 | 0.0 | 0.0 |
| 210 | Ag-387 | PR126/IR15D120 | 4242 | 31.2 | 7.2 |
| 211 | Ag-464 | PR126/IR15D120 | 2792 | 29.5 | 13.4 |
| 212 | Ag-390 | PR126/IR15D120 | 2879 | 38.3 | 14.3 |
| 213 | Ag-75 | PR126/IR15D120 | 1808 | 44.2 | 27.7 |
| 214 | Ag-264 | PR126/IR15D120 | 1397 | 7.3 | 6.3 |
| 215 | Ag-45 | PR126/IR15D120 | 3622 | 37.1 | 47.3 |
| 216 | Ag-220 | PR126/IR15D120 | 1868 | 34.7 | 26.8 |
| 217 | Ag-257 | PR126/IR15D120 | 2255 | 35.9 | 7.2 |
| 218 | Ag-366 | PR126/IR15D120 | 1915 | 45.4 | 13.4 |
| 219 | Ag-322 | PR126/IR15D120 | 2956 | 64.0 | 26.8 |
| 220 | Ag-268 | PR126/IR15D120 | 2597 | 6.9 | 25.9 |
| 221 | Ag-157 | PR126/IR15D120 | 1237 | 41.7 | 0.0 |
| 222 | Ag-399 | PR126/IR15D120 | 4688 | 72.8 | 57.1 |
| 223 | Ag-378 | PR126/IR15D120 | 3809 | 72.8 | 57.1 |
| 224 | Ag-153 | PR126/IR15D120 | 1234 | 41.7 | 26.8 |
| 225 | Ag-241 | PR126/IR15D120 | 3364 | 7.2 | 6.3 |
| 226 | Ag-331 | PR126/IR15D120 | 1576 | 21.6 | 6.3 |
| 227 | Ag-163 | PR126/IR15D120 | 3204 | 63.1 | 26.8 |
| 228 | Ag-443 | PR126/IR15D120 | 2275 | 33.8 | 13.4 |
| 229 | Ag-319 | PR126/IR15D120 | 2023 | 35.5 | 26.8 |
| 230 | Ag-158 | PR126/IR15D120 | 2131 | 20.3 | 0.0 |
| 231 | Ag-253 | PR126/IR15D120 | 1291 | 35.8 | 6.3 |
| 232 | Ag-97 | PR126/IR15D120 | 2419 | 37.3 | 20.6 |
| 233 | Ag-112 | PR126/IR15D120 | 2388 | 37.3 | 19.7 |
| 234 | Ag-431 | PR126/IR15D120 | 1699 | 12.4 | 21.5 |
| 235 | Ag-365 | PR126/IR15D120 | 1904 | 2.6 | 7.2 |
| 236 | Ag-70 | PR126/IR15D120 | 2556 | 8.5 | 0.0 |
| 237 | Ag-93 | PR126/IR15D120 | 3452 | 37.3 | 19.7 |
| 238 | Ag-468 | PR126/IR15D120 | 2192 | 8.1 | 0.0 |
| 239 | Ag-269 | PR126/IR15D120 | 2040 | 21.2 | 13.4 |
| 240 | Ag-181 | PR126/IR15D120 | 1878 | 20.3 | 0.0 |
| 241 | Ag-233 | PR126/IR15D120 | 1687 | 14.4 | 0.0 |
| 242 | Ag-449 | PR126/IR15D120 | 1550 | 19.5 | 7.2 |
| 243 | Ag-238 | PR126/IR15D120 | 2316 | 0.0 | 0.0 |
| 244 | Ag-164 | PR126/IR15D120 | 1460 | 63.1 | 19.7 |
| 245 | Ag-417 | PR126/IR15D120 | 1874 | 63.1 | 19.7 |
| 246 | Ag-306 | PR126/IR15D120 | 2851 | 35.5 | 33.1 |
| 247 | Ag-46 | PR126/IR15D120 | 2372 | 79.8 | 19.7 |
| 248 | Ag-437 | PR126/IR15D120 | 2131 | 40.9 | 0.0 |
| 249 | Ag-104 | PR126/IR15D120 | 1277 | 17.1 | 18.8 |
| 250 | Ag-149 | PR126/IR15D120 | 3206 | 13.1 | 0.0 |
| 251 | Ag-271 | PR126/IR15D120 | 2051 | 49.7 | 7.2 |
| 252 | Ag-68 | PR126/IR15D120 | 1872 | 44.2 | 25.9 |
| 253 | Ag-294 | PR126/IR15D120 | 3534 | 49.7 | 13.4 |
| 254 | Ag-27 | PR126/IR15D120 | 1981 | 50.5 | 65.2 |
| 255 | Ag-349 | PR126/IR10F365 | 1501 | 35.9 | 0.0 |
| 256 | Ag-44 | PR126/IR10F365 | 2686 | 15.7 | 20.6 |
| 257 | Ag-247 | PR126/IR10F365 | 1741 | 28.6 | 0.0 |
| 258 | Ag-419 | PR126/IR10F365 | 1618 | 0.0 | 35.7 |
| 259 | Ag-286 | PR126/IR10F365 | 1892 | 56.8 | 13.4 |
| 260 | Ag-242 | PR126/IR10F365 | 2300 | 78.7 | 53.6 |
| 261 | Ag-484 | PR126/IR10F365 | 2720 | 65.2 | 20.6 |
| 262 | Ag-177 | PR126/IR10F365 | 2360 | 34.6 | 26.8 |
| 263 | Ag-312 | PR126/IR10F365 | 1923 | 28.4 | 19.7 |
| 264 | Ag-108 | PR126/IR10F365 | 1773 | 44.4 | 12.5 |
| 265 | Ag-193 | PR126/IR10F365 | 2349 | 13.2 | 33.1 |
| 266 | Ag-438 | PR126/IR10F365 | 2125 | 14.3 | 0.0 |
| 267 | Ag-296 | PR126/IR10F365 | 2848 | 21.2 | 0.0 |
| 268 | Ag-121 | PR126/IR10F365 | 3028 | 27.4 | 13.4 |
| 269 | Ag-358 | PR126/IR10F365 | 954 | 64.4 | 13.4 |
| 270 | Ag-146 | PR126/IR10F365 | 1387 | 34.6 | 0.0 |
| 271 | Ag-203 | PR126/IR10F365 | 2215 | 20.3 | 13.4 |
| 272 | Ag-144 | PR126/IR10F365 | 3045 | 0.0 | 0.0 |
| 273 | Ag-307 | PR126/IR10F365 | 1934 | 49.8 | 26.8 |
| 274 | Ag-23 | PR126/IR10F365 | 3814 | 74.6 | 74.1 |
| 275 | Ag-341 | PR126/IR10F365 | 1009 | 0.0 | 0.0 |
| 276 | Ag-34 | PR126/IR10F365 | 1575 | 64.7 | 31.3 |
| 277 | Ag-251 | PR126/IR10F365 | 1472 | 0.0 | 0.0 |
| 278 | Ag-16 | PR126/IR10F365 | 3603 | 93.3 | 74.1 |
| 279 | Ag-147 | PR126/IR10F365 | 2073 | 41.7 | 33.1 |
| 280 | Ag-339 | PR126/IR10F365 | 2079 | 50.1 | 26.8 |
| 281 | Ag-128 | PR126/IR10F365 | 2578 | 27.4 | 22.7 |
| 282 | Ag-137 | PR126/IR10F365 | 1806 | 56.0 | 33.1 |
| 283 | Ag-244 | PR126/IR13F474 | 2319 | 0.0 | 0.0 |
| 284 | Ag-179 | PR126/IR13F474 | 2243 | 13.2 | 13.4 |
| 285 | Ag-228 | PR126/IR13F474 | 1787 | 77.4 | 40.2 |
| 286 | Ag-480 | PR126/IR13F474 | 1870 | 8.1 | 0.0 |
| 287 | Ag-481 | PR126/IR13F474 | 1313 | 29.5 | 0.0 |
| 288 | Ag-236 | PR126/IR13F474 | 2002 | 21.6 | 13.4 |
| 289 | Ag-448 | PR126/IR13F474 | 4631 | 26.7 | 0.0 |
| 290 | Ag-280 | PR126/IR13F474 | 1081 | 14.0 | 0.0 |
| 291 | Ag-227 | PR126/IR13F474 | 1162 | 0.0 | 0.0 |
| 292 | Ag-482 | PR126/IR13F474 | 2000 | 58.0 | 0.0 |
| 293 | Ag-385 | PR126/IR13F474 | 4120 | 24.0 | 20.6 |
| 294 | Ag-105 | PR126/IR13F474 | 2294 | 37.3 | 12.5 |
| 295 | Ag-56 | PR126/IR13F474 | 2389 | 1.4 | 0.0 |
| 296 | Ag-6 | PR126/IR13F474 | 2688 | 14.8 | 21.5 |
| 297 | Ag-161 | PR126/IR13F474 | 2588 | 20.3 | 6.3 |
| 298 | Ag-245 | PR126/IR13F474 | 1136 | 93.0 | 7.2 |
| 299 | Ag-49 | PR126/IR13F474 | 1884 | 37.1 | 7.2 |
| 300 | Ag-348 | PR126/IR13F474 | 2029 | 28.7 | 0.0 |
| 301 | Ag-310 | PR126/IR13F474 | 1628 | 0.0 | 0.0 |
| 302 | Ag-119 | PR126/IR13F474 | 3005 | 30.2 | 18.8 |
| 303 | Ag-400 | PR126/IR13F474 | 2852 | 13.2 | 7.2 |
| 304 | Ag-371 | PR126/IR13F474 | 1651 | 2.6 | 7.2 |
| 305 | Ag-40 | PR126/IR13F474 | 2102 | 0.0 | 7.2 |
| 306 | Ag-374 | PR126/IR13F474 | 2553 | 24.0 | 0.0 |
| 307 | Ag-396 | PR126/IR13F474 | 3424 | 13.2 | 33.1 |
| 308 | Ag-7 | PR126/IR13F474 | 2512 | 36.2 | 14.3 |
| 309 | Ag-61 | PR126/IR13F474 | 1647 | 51.4 | 33.1 |
| 310 | Ag-345 | PR126/IR13F474 | 1279 | 0.0 | 6.3 |
| 311 | Ag-176 | PR126/IR13F474 | 1593 | 0.0 | 0.0 |
| 312 | Ag-276 | PR126/IR13F474 | 1417 | 6.9 | 0.0 |
| 313 | Ag-372 | PR126/IR13F474 | 2628 | 2.6 | 7.2 |
| 314 | Ag-454 | PR126/IR13F474 | 4167 | 69.5 | 55.2 |
| 315 | Ag-2 | PR126/IR 127155-1-22 | 4256 | 69.5 | 55.2 |
| 316 | Ag-195 | PR126/IR 127155-1-22 | 3196 | 0.0 | 0.0 |
| 317 | Ag-39 | PR126/IR 127155-1-22 | 2248 | 36.2 | 7.2 |
| 318 | Ag-303 | PR126/IR 127155-1-22 | 1828 | 35.5 | 6.3 |
| 319 | Ag-282 | PR126/IR 127155-1-22 | 1178 | 64.0 | 20.6 |
| 320 | Ag-343 | PR126/IR 127155-1-22 | 1260 | 0.0 | 6.3 |
| 321 | Ag-167 | PR126/IR 127155-1-22 | 1257 | 0.0 | 0.0 |
| 322 | Ag-420 | PR126/IR 127155-1-22 | 1290 | 0.0 | 0.0 |
| 323 | Ag-191 | PR126/IR 127155-1-22 | 1471 | 48.9 | 59.8 |
| 324 | Ag-65 | PR126/IR 127155-1-22 | 2525 | 1.4 | 0.0 |
| 325 | Ag-184 | PR126/IR 127155-1-22 | 3991 | 20.3 | 0.0 |
| 326 | Ag-455 | PR126/IR 127155-1-22 | 2200 | 69.5 | 47.3 |
| 327 | Ag-377 | PR126/IR 127155-1-22 | 4870 | 66.8 | 55.2 |
| 328 | Ag-465 | PR126/IR 127155-1-22 | 3981 | 22.4 | 7.2 |
| 329 | Ag-213 | PR126/IR 127155-1-22 | 3465 | 41.7 | 7.2 |
| 330 | Ag-132 | PR126/IR 127155-1-22 | 3378 | 0.0 | 0.0 |
| 331 | Ag-350 | PR126/IR 127155-1-22 | 2746 | 0.0 | 0.0 |
| 332 | Ag-64 | PR126/IR 127155-1-22 | 1711 | 37.1 | 27.7 |
| 333 | Ag-116 | PR126/IR 93312-30-101-20-13-66-6 | 1697 | 65.8 | 25.0 |
| 334 | Ag-329 | PR126/IR 93312-30-101-20-13-66-6 | 1487 | 28.7 | 13.4 |
| 335 | Ag-428 | PR126/IR 93312-30-101-20-13-66-6 | 1103 | 40.9 | 26.8 |
| 336 | Ag-408 | PR126/IR 93312-30-101-20-13-66-6 | 2010 | 6.0 | 7.2 |
| 337 | Ag-370 | PR126/IR 93312-30-101-20-13-66-6 | 1629 | 38.3 | 26.8 |
| 338 | Ag-127 | PR126/IR 93312-30-101-20-13-66-6 | 851 | 13.1 | 0.0 |
| 339 | Ag-475 | PR126/IR 93312-30-101-20-13-66-6 | 1886 | 0.9 | 0.0 |
| 340 | Ag-323 | PR126/IR 93312-30-101-20-13-66-6 | 1792 | 42.6 | 33.1 |
| 341 | Ag-478 | PR126/IR 93312-30-101-20-13-66-6 | 1495 | 8.1 | 0.0 |
| 342 | Ag-55 | PR126/IR 93312-30-101-20-13-66-6 | 1231 | 1.4 | 0.0 |
| 343 | Ag-136 | PR126/IR 93312-30-101-20-13-66-6 | 550 | 0.0 | 0.0 |
| 344 | Ag-224 | PR126/IR 93312-30-101-20-13-66-6 | 1343 | 48.9 | 0.0 |
| 345 | Ag-426 | PR126/IR 93312-30-101-20-13-66-6 | 947 | 12.4 | 0.0 |
| 346 | Ag-337 | PR126/IR 93312-30-101-20-13-66-6 | 2062 | 0.0 | 7.2 |
| 347 | Ag-29 | PR126/IR 93312-30-101-20-13-66-6 | 2298 | 71.9 | 33.1 |
| 348 | Ag-24 | PR126/IR 93312-30-101-20-13-66-6 | 1589 | 36.2 | 26.8 |
| 349 | Ag-211 | PR126/IR 93312-30-101-20-13-66-6 | 1507 | 56.0 | 7.2 |
| 350 | Ag-171 | PR126/IR 93312-30-101-20-13-66-6 | 1663 | 7.2 | 0.0 |
| 351 | Ag-187 | PR126/IR 93312-30-101-20-13-66-6 | 1377 | 56.0 | 0.0 |
| 352 | Ag-309 | PR126/IR 93312-30-101-20-13-66-6 | 1506 | 42.7 | 34.0 |
| 353 | Ag-295 | PR126/IR 93312-30-101-20-13-66-6 | 2679 | 0.0 | 0.0 |
| 354 | Ag-102 | PR126/IR 93312-30-101-20-13-66-6 | 1536 | 30.2 | 12.5 |
| 355 | Ag-51 | PR126/IR 93312-30-101-20-13-66-6 | 1531 | 79.9 | 54.5 |
| 356 | Ag-15 | PR126/IR 93312-30-101-20-13-66-6 | 3178 | 71.9 | 67.0 |
| 357 | Ag-232 | PR126/IR 93312-30-101-20-13-66-6 | 2676 | 27.5 | 12.5 |
| 358 | Ag-299 | PR126/IR 93312-30-101-20-13-66-6 | 1364 | 6.9 | 7.2 |
| 359 | Ag-207 | PR126/IR 93312-30-101-20-13-66-6 | 2132 | 7.2 | 7.2 |
| 360 | Ag-279 | PR126/IR 93312-30-101-20-13-66-6 | 1778 | 56.8 | 20.6 |
| 361 | Ag-106 | PR126/IR 93312-30-101-20-13-66-6 | 1847 | 15.9 | 12.5 |
| 362 | Ag-204 | PR126/IR 93312-30-101-20-13-66-6 | 2582 | 27.5 | 0.0 |
| 363 | Ag-209 | PR126/IR 93312-30-101-20-13-66-6 | 1569 | 56.0 | 7.2 |
| 364 | Ag-38 | PR126/IR 93312-30-101-20-13-66-6 | 1099 | 43.3 | 33.1 |
| 365 | Ag-103 | PR126/IR 93312-30-101-20-13-66-6 | 3080 | 23.0 | 19.7 |
| 366 | Ag-138 | PR126/IR 93312-30-101-20-13-66-6 | 1142 | 55.9 | 46.5 |
| 367 | Ag-183 | PR126/IR 93312-30-101-20-13-66-6 | 1641 | 20.3 | 26.8 |
| 368 | Ag-304 | PR126/IR 93312-30-101-20-13-66-6 | 2270 | 35.5 | 14.3 |
| 369 | Ag-125 | PR126/IR 93312-30-101-20-13-66-6 | 1756 | 56.0 | 20.6 |
| 370 | Ag-451 | PR126/IR 93312-30-101-20-13-66-6 | 1631 | 5.2 | 6.3 |
| 371 | Ag-450 | PR126/IR 93312-30-101-20-13-66-6 | 2397 | 40.9 | 26.8 |
| 372 | Ag-476 | PR126/IR 93312-30-101-20-13-66-6 | 1975 | 0.9 | 7.2 |
| 373 | Ag-470 | PR126/IR 93312-30-101-20-13-66-6 | 1964 | 29.5 | 27.7 |
| 374 | Ag-62 | PR126/IR 93312-30-101-20-13-66-6 | 1522 | 51.4 | 34.0 |
| 375 | Ag-359 | PR126/IR 93312-30-101-20-13-66-6 | 779 | 7.3 | 13.4 |
| 376 | Ag-422 | PR126/IR 93312-30-101-20-13-66-6 | 2310 | 6.0 | 13.4 |
| 2021 | Environment 1 | Year 1 (2021) | 2853 | 36.0 | 24.8 |
| 2022 | Environment 2 | Year 2 (2022) | 1166 | 39.8 | 18.4 |
| 2023 | Environment 3 | Year 3 (2023) | 2499 | 37.9 | 21.6 |
| ck1 | PR126 |  | 2272 | 8.7 | 7.1 |
| ck2 | IR14D155 |  | 2034 | 75.6 | 59.9 |
| ck3 | IR15D120 |  | 1774 | 75.0 | 65.3 |
| ck4 | IR13F474 |  | 2028 | 72.2 | 65.0 |
| ck5 | IR10F365 |  | 1669 | 78.3 | 77.1 |
| ck6 | IR 127152-2-10 |  | 1638 | 66.8 | 58.9 |
| ck7 | IR 127155-1-22 |  | 1567 | 68.3 | 64.6 |
| ck8 | IR 93312-30-101-20-13-66-6 |  | 1492 | 73.3 | 58.2 |


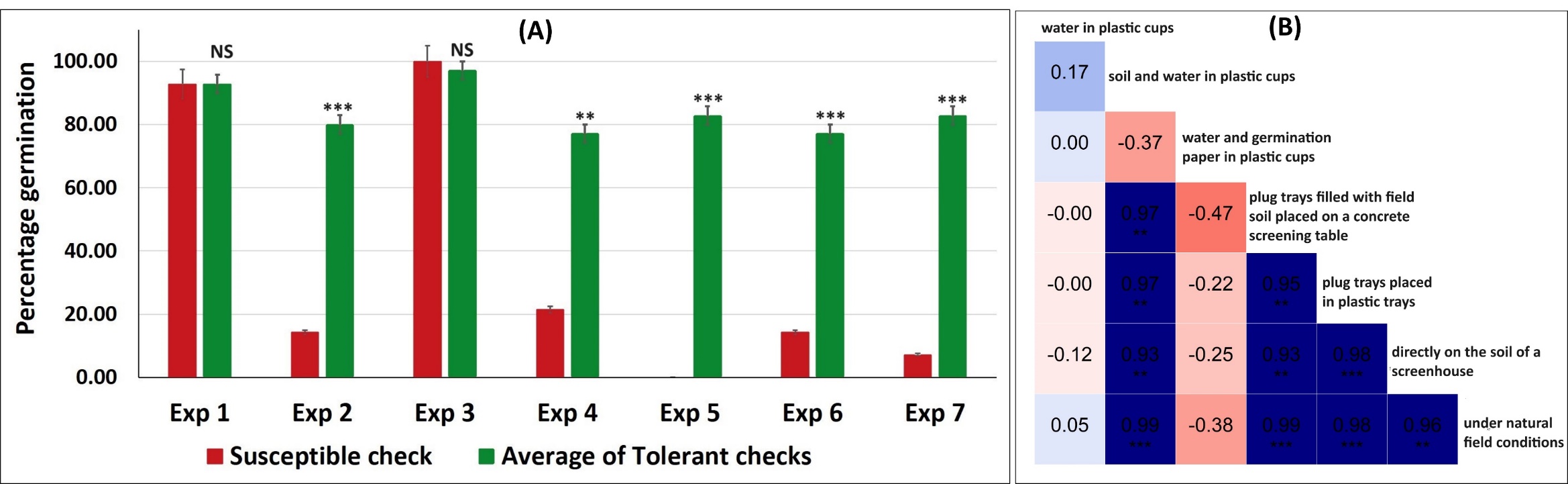


**Fig. S1** (A) The phenotypic variation between the susceptible check (PR126) and the average of the tolerant checks (IR14D155, IR13F474, IR10F365, IR127155-1-22, and IR93312-30-101-20-13-66-6) across all the seven different experiments conducted in 2020 to standardize the most effective screening method for anaerobic germination (B) Plots of Pearson’s *r-values* showing the correlation among seven different experiments that were conducted in 2020 to determine the most effective screening method for anaerobic germination. The blue color indicates positive correlation and red color indicated the negative correlation among different traits, the variation in color intensity is representing the strength of the correlation among the traits. *Significance at <5% level, **significance at <1% level, ***significance at <0.1% level.


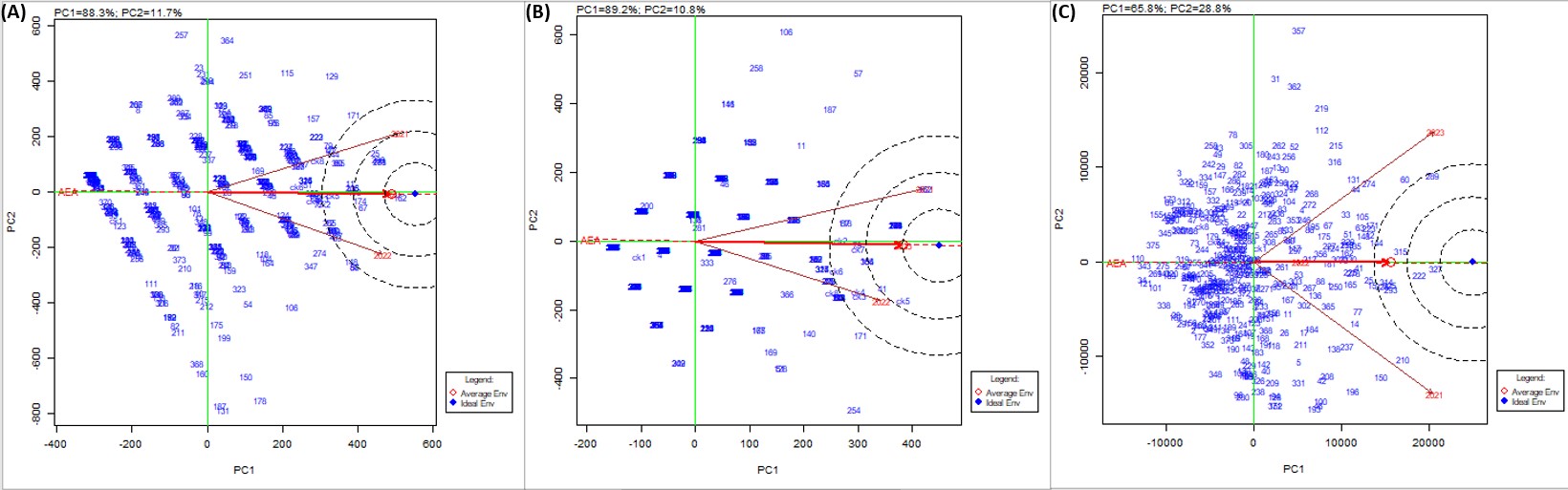
 **Fig. S2 (A)** GGE biplot showing the performance of breeding lines across years in term of percent germination in the experiment where the water was removed after 15 days **(B)** GGE biplot showing the performance of breeding lines across years in term of percent germination in the experiment where the water was removed after 21 days **(C)** GGE biplot showing the performance of breeding lines across years in term of grain yield.

The numeric code indicated in the biplot refers to each of the breeding line. The detailed information on the numeric code is provided in the Table S1
